# Creating and validating cis-regulatory maps of tissue-specific gene expression regulation

**DOI:** 10.1093/nar/gku801

**Published:** 2014-09-08

**Authors:** Timothy R. O'Connor, Timothy L. Bailey

**Affiliations:** Institute for Molecular Bioscience, The University of Queensland, Brisbane 4072, Queensland, Australia

## Abstract

Predicting which genomic regions control the transcription of a given gene is a challenge. We present a novel computational approach for creating and validating maps that associate genomic regions (cis-regulatory modules–CRMs) with genes. The method infers regulatory relationships that explain gene expression observed in a test tissue using widely available genomic data for ‘other’ tissues. To predict the regulatory targets of a CRM, we use cross-tissue correlation between histone modifications present at the CRM and expression at genes within 1 Mbp of it. To validate cis-regulatory maps, we show that they yield more accurate models of gene expression than carefully constructed control maps. These gene expression models predict observed gene expression from transcription factor binding in the CRMs linked to that gene. We show that our maps are able to identify long-range regulatory interactions and improve substantially over maps linking genes and CRMs based on either the control maps or a ‘nearest neighbor’ heuristic. Our results also show that it is essential to include CRMs predicted in multiple tissues during map-building, that H3K27ac is the most informative histone modification, and that CAGE is the most informative measure of gene expression for creating cis-regulatory maps.

## INTRODUCTION

Information controlling the patterns of gene transcription essential to the development and function of cells is encoded in the genome. Identifying the genomic regions that control the transcription of a given gene is thus of central interest. The transcription of a given gene is believed to be regulated in part by transcription factor (TF) proteins that bind to specific genomic regions called ‘cis-regulatory modules’ (CRMs), many of which may be located at great genomic distances from the gene's promoter in higher organisms.

Laboratory assays such as ChIP-seq (1) have made it possible to map the genomic locations of many TFs in a given tissue, condition, cell type or cell line (referred to hereafter as ‘tissue’), but no high-throughput assay exists for unambiguously determining which gene(s) are affected by the binding of a given TF to a given CRM in a given tissue. In the absence of such assays, ‘nearest neighbor’ rules are often used, such as assigning TF binding events to the nearest gene. The efficacy of such rules is unknown, and better computational methods for inferring ‘cis-regulatory maps’ of transcriptional regulation for each tissue are of great interest.

One computational approach for creating a cis-regulatory map is to first predict the set of CRMs active in any of a set of tissues, and then to associate them with the genes whose transcriptional expression they control. It has been shown that particular patterns of histone modifications are associated with active CRMs (2–5). Thus, data on the genomic locations of certain histone modifications in a given tissue can be used to predict the locations of CRMs in that tissue. The association of a given CRM with a given gene can be predicted using the correlation across a number of tissue types between the level of expression of the gene and the level at the CRM of a histone modification known to be indicative of active CRMs (6).

Recently, Shen *et al.* (7) created a CRM predictor for mouse embryonic stem cells that uses the presence of H3K4me1 and absence of H3K4me3 to predict enhancers (defined as CRMs not at gene promoters), which they trained using known p300 binding sites as proxies for enhancers. Yip *et al.* (6) took a different approach, using clusters of TF binding sites as proxies for CRMs, and training a method for predicting CRMs from histone modification, DNase I hypersensitivity and FAIRE data.

Yip *et al.* (6) also created a cis-regulatory map linking their predicted CRMs with genes whose expression they might regulate. To do so, they first predicted CRMs in five cell lines and merged all the CRMs (across cell lines) that overlapped. They then computed the Pearson correlation across multiple cell lines between the level of a given histone modification within a (merged) CRM and each gene within 1 Mbp (million base-pairs), assigning a link if the correlation was statistically significant for any of a panel of histone modifications.

Using a different approach, Andersson *et al.* (8) used cap analysis of gene expression (CAGE) data to both identify CRMs and to link them to target genes. They identified CRMs from short genomic regions with balanced, bi-directional, divergent transcription of short RNA molecules. They then predicted regulatory targets of CRMs identified this way from over 500 different tissues and cell lines. To predict regulatory targets, they used the correlation across tissues between short RNA transcription levels at CRMs and expression at putative transcription start site (TSS) targets within 500 Kbp of the putative CRM.

All three of these investigations that predict CRM regulatory targets lack validation of the key CRM role: that tissue-specific TF binding in the CRM regulates tissue-specific expression at the predicted target gene. A second shortcoming of these investigations is that they do not demonstrate that the mapping methodology can identify tissue-specific regulation in ‘novel’ tissues whose data were not used in creating the map. Our principal contribution is to create cis-regulatory maps using a set of tissues and show that these maps identify targets of tissue-specific TF binding in the CRMs in a novel tissue. Thus, we address both shortcomings by showing that in a novel tissue, active TF binding in a CRM corresponds to active expression at the predicted target gene and low or absent TF binding corresponds to low or absent expression at the predicted target gene. We perform this analysis through the use of regression models of gene expression.

Our computational validation approach for cis-regulatory maps is based on a regression model of gene expression. Our gene expression model predicts the RNA expression level of a gene from the amount of TF binding at all CRMs associated with the gene. We construct regression models based on different cis-regulatory maps and compare their accuracy. We reason that regression models based on more accurate cis-regulatory maps should more accurately predict gene expression. However, there are pitfalls to this validation we must address first.

Simple improvement in the accuracy of a regression model may be misleading as it can be due to problems such as overfitting or poorly chosen control cis-regulatory maps. Using too many TF parameters or commingling testing and training data can inflate the accuracy estimate of the regression models. Additionally, comparing models based on two cis-regulatory maps that model different CRM or TSS sets may result in changes in accuracy due to changes in the items involved in the map and not to changes in the quality of connections between those items. We address overfitting by using feature selection to limit the number of TF parameters of the regression model. We ensure separation of testing and training data using multiple types of cross validation. To ensure that the comparison between cis-regulatory maps is not flawed, we require that they model the same CRM and TSS sets, and only differ in the connections between the two.

Our validation procedure builds on previous work on predicting the RNA expression level of all genes from ChIP-seq or *in silico* estimates of TF binding (9,10). The regression model used in previous work predicts expression at a TSS from the binding of TFs near that TSS. In the current work, we also predict expression at a TSS from the binding of TFs in CRM regions predicted to target that TSS. We evaluate this model using the binding of TFs within either a promoter region (−500 to +200 bp of the TSS (11)) or the set of CRMs (genomic regions) associated with the TSS according to a cis-regulatory map. A crucial aspect of the present work is that we use independent data for building and validating each map. In particular, we never include the expression data used in the regression model in the data used to construct the map.

## METHODS

### Building correlation-based regulatory maps

Our cis-regulatory maps identify the genomic regions likely to be involved in the regulation of transcription of each of a subset of genes in a given tissue (‘test tissue’). The objects in our maps are annotated transcription start site (TSS) locations and CRMs. Each TSS in the map is connected by links to one or more CRMs where a link implies that the CRM affects transcription at the TSS in the test tissue.

Map creation begins with identifying CRMs from a set of tissues, including the test tissue. We use predictions of CRMs from two sources: ‘binding active regions’ (BARs) from ENCODE (6) based on histone modification data from that tissue; and enhancers from FANTOM5 (8) identified by balanced, short, bi-directional transcription. We note that other methods could be used to define CRMs including standard methods for defining enhancer regions, e.g. using the regions surrounding p300 binding sites (7), however, we do not use such data here as the cited work's data come from mouse and we constrain this study to data from humans. See Discussion for further details on potential impacts of using different types of CRMs in cis-regulatory maps.

**Figure 1.**
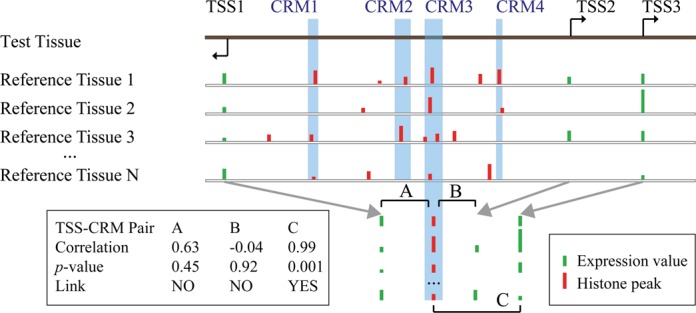
Building correlation-based cis-regulatory maps. Map links are defined by significant correlation across multiple tissues between a histone mark at a CRM and expression at a TSS. The first line in the figure illustrates the genomic locations of four CRMs and three nearby TSSs. The next four lines illustrate the aligned locations of ChIP-seq peaks for a particular histone modification (red bars) and the measured RNA expression at the three TSSs (green bars) in *N* comparative (‘reference’) tissues. The arrows and left inset illustrate the computation of the Pearson correlation coefficient, its *P*-value and the presence of a link in the map at a stringency of *P* = 10^−2^ for three (out of 12 possible) CRM-TSS pairs.

To create links between annotated TSSs and either ENCODE or FANTOM5 CRMs, we look at the correlation between the expression at the TSS and the histone state of a particular CRM across a number of different tissues (Figure 1). Here our approach differs from that of (6) in that we do not amalgamate clusters of overlapping CRMs from different tissues into a single, tissue-nonspecific CRM, but rather keep a non-overlapping subset of tissue-specific CRMs from these clusters (see Supplementary Methods for details). Importantly, we use not only the CRMs active in the test tissue, but also those that are inactive, looking for CRMs where the presence of a histone modification in that region of the genome is strongly correlated with expression at a given TSS across multiple tissues. Essentially, we ‘project’ the CRM coordinates onto the histone ‘tracks’ for the comparative tissue types (Figure 1).

Our map-building approach has several benefits. First, because we do not amalgamate CRMs predicted in different tissues, our CRMs do not include extraneous genomic regions added in the amalgamation nor combine two CRMs with potentially different activity into a single CRM. Second, including (but not amalgamating) CRMs from multiple tissues (five from ENCODE and more than 500 from FANTOM5) in the map-building process increases the quality of the map by providing more evidence of correlation between active and inactive enhancers and changes in expression at target genes. Third, our approach allows us to validate the reliability of the map by leaving out the test tissue in the correlation step, and then measuring the accuracy of a map-based regression model of gene expression in the left-out tissue. Since we do not use expression data from the test tissue in building the map, prediction of this expression data from TF ChIP-seq data is an unbiased validation approach. The fact that we include CRMs from multiple tissues in the map improves our ability to validate the map using regression. This is due to the fact that many CRMs from tissues other than the left-out one are likely to be inactive and unbound by any TF in that tissue, and the genes linked to them in the map are likely to have low expression. This provides additional contrast for more accurate regression.

### Validating correlation-based regulatory maps

To validate our approach for building correlation-based regulatory maps, we employ a cross-validated LASSO linear regression model (12,13) that predicts gene expression from TF binding. Our new map-based expression model, which is based on prior models (e.g. (9) and (10)), predicts transcription at each TSS from the TF binding profile of each of its map-associated CRMs. We compare the ability of this model to explain the variance in the expression in the map tissue with that of a randomly sampled CRM-TSS map model with similar properties to the correlation-based map (see Supplementary Methods for details). Importantly, our map-validation approach avoids circularity because *none* of the data used to validate it (TF binding and expression data for the test tissue) was used to construct it (histone and expression data for *other* tissues). We recommend using expression data from the test tissue when constructing the final map, as we expect that this will produce a more accurate map. Here we report only the accuracy using maps constructed with expression data from the test tissue left out because improvements in the accuracy of models containing data from the test tissue, while more reflective of the biology, would be inflated estimates due to data circularity. The reported accuracy values form a pessimistic lower-bound for the true accuracy of a map built using expression data from the test tissue.

To summarize, our validation approach is as follows. Using either the ENCODE or FANTOM5 CRMs, we create a correlation-based map omitting the histone and expression data for a particular tissue on which we will test the map (the ‘test’ tissue). Then, using TF binding and expression data only from the test tissue, we fit a map-based LASSO regression model that predicts expression at a TSS from measures of TF binding within any of its map-associated CRMs. (See Supplementary Methods for details.) Next, we estimate the accuracy of the regression model using the mean and standard error of the regression fit (*R*^2^) across each fold of the LASSO cross-validation. (See Supplementary Methods for details on fitting and scoring the regression model.) Finally, we calculate this same LASSO fit using a set of carefully constructed control maps, whose links are based on random sampling, and compare this fit to that derived from the correlation-based cis-regulatory map. Details of the map sampling method are described in the next section.

### Sampled regulatory maps for comparison purposes

To validate our correlation-based regulatory map, we need to show that the fit of the regression models based on cis-regulatory maps comes from the particular CRM-TSS links that the cross-tissue correlation selects and not from some other property of the map. Thus, we also build regression models using a simplified map that we call a ‘sampled control’ map.

We construct the sampled control map to be the same as the correlation-based map with respect to five properties, differing from it only in the links that it contains. Thus, the sampled maps contain exactly the same TSSs as the particular correlation-based map in order for accuracy comparisons to be fair. This is necessary because the expression of some TSSs might be inherently easier to model with regression than others. Additionally, each sampled map includes the same number of links as the correlation-based map. Furthermore, the sampled maps have a similar distribution of the number of CRMs connected to each TSS in the map. Thus, both types of maps have the same connectivity properties. Next, the sampled maps have a similar distribution of the relative position of the CRMs to the TSSs (both distance and direction), eliminating distance-dependent effects of TF binding on expression as a source of change between the two maps. Finally, the sampled maps contain the same set of CRMs that were considered during creation of the correlation-based map (see Supplemental Methods for details on when CRMs can be mapped). In order to account for the variation in sampled maps, we always report the average and standard error of data from 10 independently sampled maps.

## RESULTS

### Cis-regulatory maps identify tissue-specific, long-range transcription factor regulatory targets

**Figure 2.**
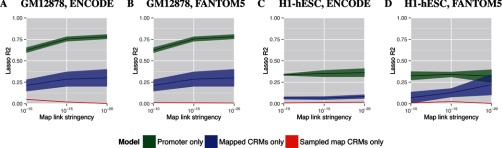
Accuracy of cis-regulatory maps built with ENCODE or FANTOM5 CRMs using two different cell lines (GM12878 and H1-hESC) as test tissues. Each plot shows how the accuracy (explained variance, vertical axis) of models of gene expression based on cis-regulatory maps changes as a function of the link stringency (horizontal axis) of the H3K27ac-CAGE histone-expression correlation of the CRM-TSS links in the map. The green and blue curves and areas show the mean and standard error of the LASSO *R*^2^ for expression models using TF binding in either the promoters or CRMs of the map TSS set, respectively. The red curve shows the mean and standard error of the LASSO *R*^2^ for 10 expression models based on 10 sampled control maps. Panels (**A**) and (**B**) show accuracy of models of CAGE expression in lymphoblastoid cell line GM12878 using ENCODE and FANTOM5 CRMs, respectively. Panels (**C**) and (**D**) show accuracy of models of CAGE expression in stem cell line H1-hESC using ENCODE and FANTOM5 CRMs, respectively.

We examine the ability of cis-regulatory maps to identify tissue-specific, long-range TF targeting in a single test tissue, GM12878, a lymphoblastoid cell line. We construct cis-regulatory maps for this tissue using either BARs from ENCODE (6) or enhancers from FANTOM5 (8) as the initial set of CRMs. We then compute the significance of all possible CRM-TSS pairs (maximum distance 1 Mbp) using the correlation across 11 other tissues between H3K27ac histone marks at the CRM and CAGE expression data at the TSS as described in Methods. By thresholding the significance of the CRM-TSS links, we create a series of correlation-based maps with decreasing numbers of TSSs, CRMs and links as the statistical stringency for links increases (Figure 2A, B).

In addition to the correlation-based map, at each threshold we also construct 10 maps using sampled links as a control. We carefully construct this control so that the only substantive difference between the correlation map and the sampled maps are the particular links included in the map. Thus, the sampled control maps have the same TSS set, the same number of links (and thus CRMs), a similar distribution of CRM positions relative to the TSS (Supplementary Figure S2) and a similar distribution of the number of CRMs associated with each TSS (Supplementary Figure S5).

For each map stringency, we fit the LASSO regression model to both the correlation and sampled maps. As the link stringency increases, the fraction of explained variance increases in correlation map based regression models that predict CAGE expression data in GM12878 cells from ChIP-seq binding data for 53 TFs (Figure 2A, B, blue curve). The TF binding in CRM regions in the control maps explain virtually no variance in gene expression (Figure 2A, B, red curve). The ability of the TF binding in CRMs to explain expression of the predicted CRM regulatory targets and the lack of ability for the TF binding in the CRMs linked to targets using sampling shows that the correlation mapping method identifies biologically relevant regulatory targets. We next look at this phenomenon across multiple tissues to see if this ability to explain expression is a general property of cis-regulatory maps.

### Cis-regulatory maps explain gene expression in multiple test tissues

To further evaluate our map-building procedure, we repeat the map building and validation process used for GM12878 to make correlation-based maps for four additional test tissues: H1-hESC (Figure 2C, D); HeLa-S3, Hep-G2 and K562 (Supplementary Figure S1). As before, we use CAGE expression data and H3K27ac, CRMs from ENCODE or FANTOM5 and do not use any data from the test tissue (other than the CRMs) in building the map. For each of the five test tissues, the correlation-based maps at the highest link stringency contain comparable numbers of TSSs, CRMs and links. For maps using ENCODE CRMs, these counts are: 1,471–1,938 TSSs, 18,080–22,474 CRMs and 40,027–55,001 links. For maps using FANTOM5 CRMs, they are 869–1,199, 1,075–1,688, 2,530–4,627, respectively. The median number of CRMs linked to a given TSS is 17–20 for ENCODE and two for FANTOM5. We report the explained variance (LASSO *R*^2^) for regression models based on the correlation map, the sampled map and the promoter-only model (Figure 2, Supplementary Figure S1).

For all five test tissues, the correlation-based maps result in superior models of gene expression compared with sampled-map control models. The improvement is particularly large for the FANTOM5-based cis-regulatory maps. The explained variance increases from about 0.1 to over 0.3 for the correlation map based model at link stringency of 10^−20^ using FANTOM5 enhancers. This result further supports the hypothesis that the correlation-based map-building process generalizes to identify *bona fide* regulatory links between CRMs and TSSs in novel tissues. Next, we look at the use of different types of RNA expression measurement in creating maps for these same tissues to see if the differences in these data sources have an impact on our ability to identify CRM-TSS links.

### CAGE expression identifies CRM targets better than poly A+ or poly A- RNA-seq

We have shown thus far that correlation-based cis-regulatory maps created using CAGE expression data capture regulatory links between TSSs and CRMs. We now show that this is also true for maps created using two other types of RNA expression data (poly A+ and poly A- RNA-seq). As before, we create and validate maps for five test tissues (GM12878, H1-hESC, HeLa-S3, Hep-G2 and K562), but using different types of expression data (poly A+ and poly A- RNA-seq). For each type of RNA data, we report the mean and standard error of the explained variance (*R*^2^) across the five test tissues.

**Figure 3.**
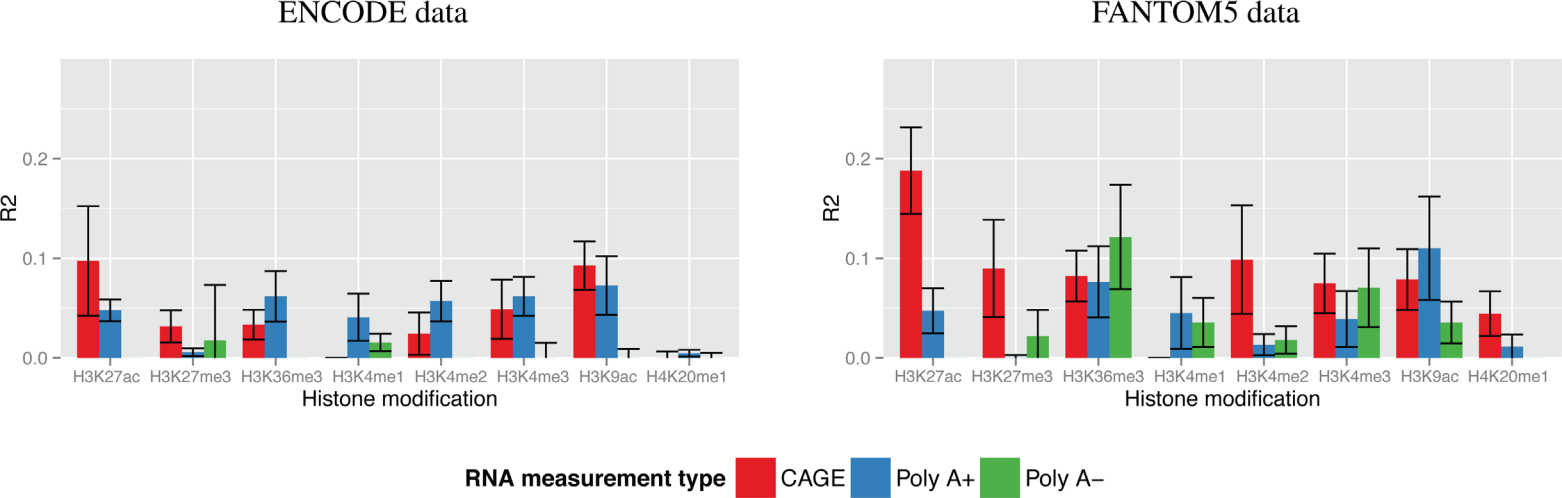
Accuracy of cis-regulatory models for different histone modifications† and types of RNA expression data, averaged over five test tissues. Each bar shows the average and standard error of explained variance (vertical axis) of gene expression models for five tissues, each built and validated using the given histone modification (horizontal axis) and RNA data type. Each group of bars shows the average explained variance for each type of RNA data for a given histone modification. All data in this plot use maps with a correlation-map link stringency of (θ = 10^−20^). The left and right panels show data using CRMs from Yip *et al.* (6) and Andersson *et al.* (8), respectively. †Data missing for H3K4me1 using CAGE RNA measurements.

Only the regression models built using H3K27ac-CAGE and H3K27ac-poly A+ correlation-based maps for each of five cell types substantial explained variance in the regression models (Figure 3A, B, H3K27ac bars). The CAGE regression models have an *R^2^*≈ 0.10 and *R^2^* ≈ 0.18 using ENCODE and FANTOM5 CRMs, respectively. The poly A+ regression models have an *R^2^* ≈ 0.05 for both CRM sources. There is virtually no explained variance using the poly A- data (*R^2^*≈ 0.0). Note that none of the sampled control maps have any explanatory variance (*R^2^* ≈ 0.0, Supplementary Figures S6 and S7, red curve). In fact, in 28 of 30 cases we test across CRM sources and link stringencies, models built using H3K27ac-CAGE correlation-based maps are more accurate then sampled map models (Supplementary Figures S6 and S7, blue curves versus red curves). We next look at the promoter-only-based regression to set our expectation for how well the expression at the target TSSs can be modeled.

The accuracy of promoter-based models of expression of cis-regulatory map TSS targets varies significantly depending on the type of RNA expression data used to construct the map. The CAGE-based expression models show highly explained variance (*R^2^* ≈ 0.30 to *R^2^* ≈ 0.80) across tissues andCRM sources, whereas poly A+ does not exceed *R^2^* ≈ 0.25 and poly A- exceeds *R^2^* ≈ 0.10 in only 1 out of 10 cases (Supplementary Figures S6 and S7, green curves). The drops in ability of cis-regulatory maps to explain poly A+ or poly A- maps using H3K27ac may be primarily due to the inherent difficulty in modeling those expression types and not due a deficiency in the mapping methodology. However, because the different RNA measurements may reflect different transcription processes (PolII versus PolIII) or different quantification methods (aggregate TSS activity versus assembled transcripts), H3K27ac may not be suitable to identify CRM-TSS links with expression sources other than CAGE. Thus, we next turn to analyzing alternate histone data sources for building cis-regulatory maps.

### Active enhancer marker H3K27ac best identifies CRM targets

To evaluate the role of histone marks in identifying long-range CRM regulatory targets via cross-tissue correlation, we constructed maps for the five tissues mentioned above using each of six or seven other histone markers (Table 1). We performed the same cross-validated LASSO regression between TF binding in CRMs and their predicted targets for each map and its corresponding sampled maps. We report the mean and standard error of this fit at the highest map stringency (*P* < 10^−20^) for both ENCODE and FANTOM5 CRMs for each RNA expression data source. Below, we examine three types of histone modifications and their ability to identify CRM targets: enhancer related (H3K27ac and H3K27me3), promoter related (H3K4me3 and H3K9ac) and gene body related (H3K26me3).

**Table 1.** Data sources for map generation and validation

*Data type*	*Values*
Test Tissues (TFs)	GM12878 (53), H1-hESC (22), HeLa-S3 (56), Hep-G2 (39), K562 (77)
Reference Tissues†	A549, AG04450, BJ, GM12878, H1-hesc, HeLa-S3, Hep-G2, HMEC, HSMM, HUVEC, K562
	MCF-7, NHEK, NHLF, SK-N-SH_RA
RNA Expression Data	CAGE, poly A+ RNA-seq, poly A- RNA-seq
Histone Modifications	H3K27ac, H3K27me3, H3K36me3, H3K4me1‡, H3K4me2, H3K4me3, H3K9ac, H4K20me1
	

This table lists the cell lines that we omit from constructing maps (Test Tissues), the comparative cell lines (Reference Tissues) and histone modification data (Histone Modifications) used in map construction and the types of RNA expression data (RNA Expression Data) we use in map construction and validation. The number of transcription factors used in map validation is shown in parentheses after the cell line name. For a full list of expression data availability for each tissue see Supplementary Table S2. †Not all tissues have all types of expression data. ‡H3K4me1-CAGE tissue set is insufficient for correlation mapping.

Modifications to histone 3 lysine 27 are associated with enhancer activation and inactivation (14). Not surprisingly, the marker of active enhancers (H3K27ac) best identifies CRM-TSS regulatory links when correlated with CAGE expression (Figure 3A, B, red H3K27ac bars). The tri-methylation of this same lysine residue shows much lower ability to identify CRM-TSS links. We also see that the CRMs with significant H3K27me3-CAGE correlation are distributed evenly over the ±1 Mbp region we examine indicating these links are more likely due to random chance (Supplementary Figure S3B versus A). Next, we see that promoter-associated histone modifications also identify CRM-TSS regulatory links, producing the best poly A+ derived cis-regulatory maps (H3K9ac). Given that these histone marks are promoter associated, the cis-regulatory maps may be identifying alternate promoters that regulate genes’ expression rather than CRMs. Alternately, if CRMs are in close physical proximity to the promoter they regulate, they may be incidentally cross-linked with the promoter's histones during the ChIP-seq measurement. Finally, H3K36me3, whose presence in gene bodies strongly correlates with expression of those genes (15), also successfully identifies CRM-TSS links. Since H3K36me3 is not normally thought of as a mark associated with enhancers, this result may seem counter-intuitive. However, we observe that many CRMs are located within gene bodies, so our map-building process is able to leverage the known correlation between H3K36me3 and gene expression to identify CRM-TSS links. We next look at the effects of the different CRM sources in cis-regulatory map construction due the general improvement in regression fit when using FANTOM5 CRMs.

### Comparison of CRM sources

In each of the five tissues examined in Figure 2, Supplementary Figure S1, the models of gene expression based on maps with FANTOM5 CRMs always outperform those based on maps with ENCODE CRMs. We examined the average fit of gene expression models across the five tissues for each histone modification for both of these CRM sets. For six out of seven histone modifications used for map construction with CAGE expression, the models with FANTOM5 CRMs have better fit than the those with ENCODE CRMs (Figure 3B versus A, red bars). When we repeated this experiment using different RNA expression data sources (poly A+ and poly A-), the models using FANTOM5 CRMs were equivalent to or better than the models based on ENCODE CRMs in six of eight and seven of eight cases (Figure 3, blue and green bars), respectively. The improved fit of the models with FANTOM5 CRMs provides strong evidence that these CRMs contain more regulatory information than the ENCODE CRMs. We next examine the principal differences between these CRM sources.

We used 553,910 distinct, non-overlapping ENCODE CRMs from five tissue sources and 43,011 FANTOM5 CRMs from over 500 tissue sources. The ENCODE CRMs are based on predictions of high TF binding using histone ChIP-seq, FAIRE and DNAse hypersensitivity data whereas the FANTOM5 CRMs are based on balanced, bi-directional, short RNA transcription as measured by CAGE. Table 2A and B shows that the ENCODE CRM set—being an order of magnitude larger than the FANTOM5 set—produces maps with over an order of magnitude more links and links per TSS at the same link stringency. Despite the lower number of CRMs and links, the FANTOM5-based maps identify TF binding that explains expression as well or better than the ENCODE based maps in 13 out of 15 cases (Supplementary Figure S7 versus S6), indicating that CRMs from a larger set of tissues model gene expression more accurately, and that the FANTOM5 CRMs identify regions whose TF binding is much more likely to have regulatory function. Because proximity is commonly used to predict which CRMs target a gene, we next examine the FANTOM5 CRMs in more detail by comparing cis-regulatory maps made using cross-tissue correlation and nearest-neighbor (NN) methods of predicting regulatory targets.

**Table 2.** Properties of cis-regulatory maps built from H3K27ac-CAGE correlation and different CRM sources using a lymphoblastoid cell line (GM12878) as a test tissue

**A**	**ENCODE**				
θ	*Links*	*CRMs*	*TSSs*	*Genes*	*Links/TSS (median)*
10^−10^	73 585	30 045	2712	1341	27.1 (16)
10^−15^	54 189	23 561	1948	977	27.8 (18)
10^−20^	40 027	18 080	1528	765	26.2 (17)

**B**	**FANTOM5**				
**θ**	***Links***	***CRMs***	***TSSs***	***Genes***	***Links/TSS (median)***
10^−10^	4280	1731	1556	779	2.8 (2)
10^−15^	3223	1353	1122	581	2.9 (2)
10^−20^	2530	1075	869	455	2.9 (2)
					

These tables show the numbers of CRM-TSS links, CRMs, TSSs, genes and average (and median) links per TSS in the correlation-based maps at the given link stringency (θ). Tables **(A**) **and (B)** show these properties for maps created using CRMs from ENCODE and FANTOM5, respectively. All correlation maps here are created using H3K27ac-CAGE correlation omitting data from GM12878.

### Correlation maps identify biologically relevant nearest-neighbor links

A common heuristic to identify which CRM targets a particular TSS is to assign the nearest-neighbor CRM to that TSS. We next compare the correlation-based maps to maps built using this heuristic. To do so, we look at the nearest-neighbor maps for the TSS set in the map built using FANTOM5 CRMs and H3K27ac-CAGE correlation with a link stringency *P* < 10^−20^, omitting data from GM12878. We first note that nearest neighbors only account for a small fraction of the correlation-based map. For instance, the links of CRMs among the first three nearest to each TSS only constitute roughly one third of those in the correlation-based map, despite having roughly the same number of overall links (Supplementary Figure S9). This lack of nearest-neighbor CRMs in the correlation-based maps implies that many of the CRMs that regulate a gene are not the ones proximal to that gene.

**Figure 4.**
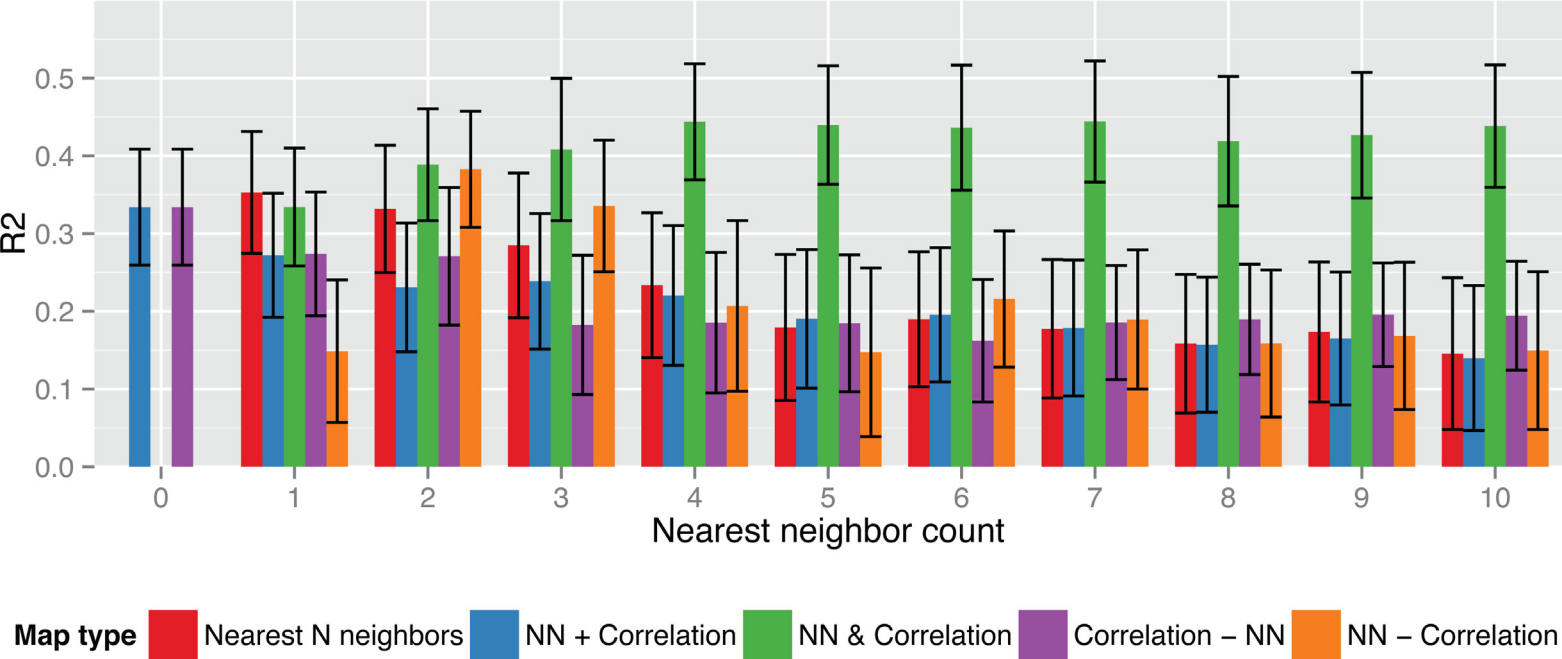
Accuracy of H3K27ac-CAGE correlation- and NN-based cis-regulatory models in GM12878. Each bar shows the average and standard error of explained variance (vertical axis) of gene expression models for GM12878 using either correlation or NN, or some combination thereof. Each group of bars shows accuracies of increasing numbers of nearest neighbors (horizontal axis). All data in this plot that with correlation-based links use a link stringency 10^−20^. The red bars show data using only the NN CRMs. The blue bars show data using any CRM-TSS link in either map type. Note that when zero nearest neighbors are used, this is identical to only the correlation-based map. The green bars show data using only CRM-TSS links that are both a NN and have high cross-tissue correlation. The purple bars show data using only the correlation-based CRM-TSS links that are ‘not’ nearest neighbors. Finally, the orange bars show data using only the NN CRM-TSS links that are not in correlation-based map.

Next we examine the fit of TF-expression regression models on sets of links that are in both, either, or the difference between the nearest-neighbor and correlation-based maps (Figure 4). We note that the single nearest-neighbor map produces strong regression fit (*R*^2^ ≈ 0.35). However, if we remove those links from the nearest-neighbor map that are identified as regulatory by the correlation-based map, the fit drops significantly (*R*^2^ ≈ 0.15). This drop implies that the correlation-based map has distinguished which nearest-neighbor links are regulatory and that upon removing them, the nearest-neighbor map is depleted of regulatory relationships. Furthermore, if we look at the links in the intersection between the correlation-based map and nearest-neighbor map, regression models based on this intersection consistently produce a fit as good as or better than the correlation-based map (*R*^2^ ≈ 0.33 to 0.45). This high fit also confirms that the correlation-based map identifies which nearest-neighbor links are regulatory and also implies that regulatory CRMs closer to the TSS may have more influence on expression at that TSS.

### Characteristics of CRM targets

We have thus far only looked at the traits of the CRM-TSS links and have not examined any traits of the target TSSs and nor whether those involved in CRM-TSS links are different from those that are not. Thus, next we look at the characteristics of the gene targets in CRM-TSS maps. In particular, we look at their promoter characteristics, whether they are in any known genomic blocks enriched in specific types of CRM-TSS regulation, and their functional enrichment. For simplicity, througout this section we will use the cis-regulatory map built with H3K27ac-CAGE correlation that omits GM12878 at a link stringency of 10^−20^.

**Figure 5.**
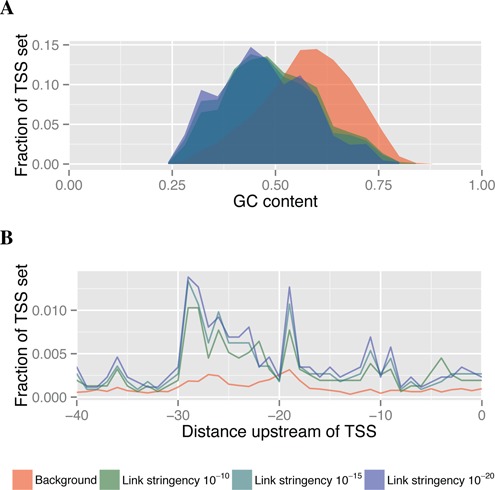
GC content and TATA characteristics of promoters of FANTOM5 CRM target TSSs for cis-regulatory maps built with H3K27ac-CAGE correlation omitting GM12878. Panel (**A**) shows the distribution of the fraction TSSs (vertical axis) with a given GC-content (horizontal axis) in the 500 bp upstream of the TSS. Panel (**B**) shows the fraction of TSSs with a TATA tetramer (vertical axis) ending at the given position upstream (horizontal axis, measured in bp) in the 40 bp upstream of the TSS. The red region or curve shows the backround distribution of the given measurement for all TSSs considered for correlation-based mapping. The green to blue regions/curves show the distribution for promoters of TSSs in cis-regulatory maps with increasing link stringency.

Previous CRM mapping studies have reported that CRM target promoters are GC-depleted (8). We tested if our predicted targets have this property by comparing the distribution of GC-content of all promoters (using the 500 bp upstream of the TSS) that were considered for correlation mapping and those in an H3K27ac-CAGE cis-regulatory map (with FANTOM5 CRMs) for each link stringency. Consistent with previous results (8), we see that the GC-content of promoters of TSS targets is depleted relative to the background, and that it decreases with increasing link stringency (Figure 5A).

Similarly, we investigate the TATA dependency of CRM target TSSs by examining the fraction of a TSS set with TATA tetramers at a given position upstream of the TSS. We use the same background and cis-regulatory map sets of TSS targets as above. We use this tetramer rather than the full TATA-box motif (16) to get a clearer difference because the TATA motif counts are very low in both the background and CRM target promoter sets (data not shown). Compared with the background promoter set, TSSs in the map are four to five times more likely to contain the TATA tetramer located approximately 30 bp upstream of the TSS (Figure 5B). This increase supports the hypothesis that genes regulated by CRMs are more likely to be TATA dependent.

Genomic regulatory blocks (GRBs) (17,18) and super enhancers (SEs) (19,20) are genomic regions enriched in CRM-TSS regulatory pairs. GRBs are identified by clusters of ultra-conserved non-coding elements, whereas SEs are identified by large clusters of enhancer-like markers accompanied by specific cell-identity related TF binding. We compare a cis-regulatory map to these regions to determine if these genes may be involved in the same type of CRM-TSS regulation as revealed by the cross-tissue correlation mapping method (Supplementary Table S6).

We find that the fraction of a selected cis-regulatory map's links with both the CRM and the TSS in either a GRB or SE does not exceed 1% of the total links in the map. The fraction of GRBs found overlapping a link in a cis-regulatory map does not exceed 10% and the number of SEs found overlapping a link does not exceed 2%. The low correspondence between these genomic regions and our cis-regulatory maps is likely due to the lack of CRMs and TSSs in these regions that meet the criteria for correlation-based mapping.

We examine two genes from the H3K27ac-CAGE correlation map in Supplementary Figure S10. We show the FANTOM5 CRMs we tested as putatively targeting these genes, the PCC value from the mapping process and the *P*-value to determine which CRMs we predicted as targeting these genes. The first gene, LRRTM3, is a transmembrane protein with three CRMs within ±1 Mbp of its TSS, one of which we predict targets the gene, despite being located approximately 950 Kbp downstream of the TSS. The second gene, MYCN, is a transcription factor with 33 CRMs within ±1 Mbp of its TSS, of which we predict 22 (six upstream and 16 downstream) CRMs target that gene. Given the membrane and regulatory functions of these genes we examined the enriched cellular components and biological processes in the map (Supplementary Table S7) using the DAVID tool (21,22) (see Supplemental Methods for details). Interestingly, the most highly enriched GO terms are related to the extracellular region, indicating that CRM-based regulation is important in inter-cellular or environmental interactions and responses. To see if TSSs with different CRM-TSS connectivity are implicated in different biological functions, we compared the singly-linked TSS set with the multiply-linked TSS set. There were no terms significantly (*P* < 0.01) enriched in either set compared to the other (data not shown), so our results do not identify any biological functions, processes or components that are associated with singly- or multiply-linked genes.

## DISCUSSION

This study provides several important biological and computational contributions. We provide a validation of cis-regulatory maps that directly examines the most biologically pertinent regulatory actors–transcription factors, and show that these maps do identify novel, long-range TF regulatory targets. Furthermore, we provide new insights into the importance of the data sources used in building these maps. First, that the TSS specificity of CAGE expression aids in identifying CRM targets. Next, that H3K27ac provides the strongest evidence of CRM targets. Finally, that CRM sources from a larger number of tissue sources (FANTOM5) more clearly show the correspondence between TF binding and expression at target TSSs. Additionally, this study is the first bioinformatic validation of cis-regulatory maps to avoid data circularity by using validation data from completely distinct tissues from those used to generate the map.

While our validation approach creates cis-regulatory maps that omit data from a test tissue to ensure independent data for validation, our the results imply that using all available data results in more accurate and useful maps. In fact, our results suggest that it might be possible to construct a general cis-regulatory map for a given organism whose accuracy would improve as data from increasingly diverse tissue types is included in its construction.

In order to validate cis-regulatory maps, we found it essential to include CRMs from multiple tissues. This is due to the fact that maps that only include enhancers active in the test tissue do not provide crucial information that is present when CRMs inactive in the test tissue are included in the map. In particular, these inactive CRMs allow the regression to ‘learn’ that ‘lack’ of TF binding is correlated with ‘lack’ of expression of the linked gene.

While we have described a strong computational validation of cis-regulatory maps, the ideal way to validate such a prediction method would be to measure its accuracy at predicting a known set of CRM-TSS interactions. However, proving that a particular genomic region is or is not involved in controlling transcription at a TSS is extremely difficult experimentally, and to our knowledge, no curated lists of CRM-TSS pairs and non-pairs exist yet for any tissue. Circumstantial evidence would be provided if the TSS and CRM could be shown to be physically in contact, but the resolution of methods for predicting this genome-wide (such as Hi-C (23)) is currently too low to be of use in mammals. Current Hi-C interaction maps for human can only predict whether two 20 Kbp genomic regions might be in contact. ChIA-PET (24) offers another means of identifying CRM-TSS relationships, but only identifies those mediated by a single TF. We have circumvented these difficulties by showing that the CRM-TSS pairs identified by our cis-regulatory maps are predictive of gene expression. Our results show that the predictive power of models of expression based on these CRM-TSS links increases with the statistical confidence assigned to the links by our map-building approach.

Regulatory maps constructed using the histone-expression correlation approach have many uses. First, they direct the experimentalist toward genomic regions are likely to be involved in regulation the transcription of particular transcripts. This use should facilitate the identification of key regulatory regions and mutations. Overlaying TF ChIP-seq data from a tissue with the CRMs in a map and following the map links to TSSs could be used to predict novel regulatory targets of the TF, especially as a number of TFs appear to preferentially bind to enhancers (5). Second, the CRMs linked with high statistical confidence to a given TSS can be searched bioinformatically for enrichment of transcription factor binding sites (25–27) even when TF ChIP-seq data in the mapped tissue is not available.

The histone-expression correlation approach for building cis-regulatory maps has several advantages. First, it leverages the regulatory information present in widely available (and growing) data from sources such as the ENCODE project (28). This data can (and should) include tissues or cell types that differ substantially from a tissue of interest. This method will also gain statistical power as histone and expression data for more tissues become available. Second, our map-building method does not require expression or TF binding data for the tissue of interest, only a way to predict CRMs in that tissue. However, given the breadth of data available in the FANTOM5 CRM set, predicting CRMs in a tissue of interest may not be needed simply because such a large number of tissues and conditions were covered in that study.

We used CRMs predicted by Yip *et al.* (6) from histone, DNase I and FAIRE data and Andersson *et al.* (8) from balanced, bi-directional CAGE, but CRMs predicted only on H3K4me1 and H3K4me3 ChIP-seq (7) or with other methods could be used with our approach. However, the approach of CRM identification may be crucial in identifying long-range TF targeting. We make this caution for two reasons. First, there are many types of CRMs, such as enhancers and repressors, which may behave and map to targets differentaly. Second, detection of multiple enhancers in a cluster may indicate a single super enhancer (19) which may need to be mapped to a target as a single entity rather than by its constituent enhancers. We look into the next caution in further detail.

The ENCODE CRMs, which are based on predictions of high TF binding activity, model gene expression of their targets rather poorly, whereas the FANTOM5 CRMs perform much better. We have discussed the possibility that this may be due in part to the number of tissues involved in the FANTOM5 set, which allows the regression to fit more inactive enhancer's TF binding to inactive expression at the target TF. An additional hypothesis is that assumption underlying the ENCODE CRM prediction method—that high TF binding density implies regulatory activity—may be incorrect. The hypothesis that this assumption is false is further supported by the combination of a vast number of CRMs and low regression accuracy in the ENCODE set relative to the FANTOM5 set. Also, in contrast to the ENCODE CRMs, there is active transcription at the FANTOM5 CRMs, indicating the presence of PolII transcription which strongly indicates these CRMs are involved in regulatory processes. It is our conjecture that much of the TF binding that identifies ENCODE CRMs is not regulatory binding, but perhaps may simply be random binding or ‘parking’ of TFs for potential regulatory use. To investigate which hypothesis has better support, a subset of FANTOM5 CRMs from only five tissues could be used to control for the influence of the number of tissues on the gene expression models. This control would isolate the regulatory relevance of the CRMs as the differentiating factor between the ENCODE and FANTOM5 CRM sets. At the time of writing, subsets of FANTOM5 CRMs from individual tissues were not available so we could not perform this analysis to see which hypothesis has better support.

Future work will explore several directions. First, our correlation-based method of identifying CRM-TSS relationships, like the method of Yip *et al.* (6) from which it is derived, uses the Pearson correlation coefficient. This implicitly assumes that there is a collinear relationship between a single histone modification at the CRM and the expression at the TSS. We intend to evaluate alternate correlation measures such as the Spearman correlation coefficient and mutual information, as well as Bayesian methods that integrate multiple histone sources simultaneously. Additionally, it might make sense to evaluate the roles of different CRM-binding TFs based on their importance in this regression model or more complicated models such as SVR. Such an investigation may provide evidence for distinct types of regulatory relationships, such as the sets of CRM-related TFs identified in genome segmentation studies (29,30). For example, binding at a CRM by a particular TF might have a different effect on expression depending on whether the CRM is marked by H3K4me3 or H3K27ac. Finally, we will explore eliminating the need to predict CRMs at all, and test the ability of a correlation-based approach to directly predict regulatory links between TF binding events and TSSs. This would enable researchers to link ChIP-seq peaks to target genes.

## SUPPLEMENTARY DATA

Supplementary data are available at NAR Online.

SUPPLEMENTARY DATA
