# Supplementary material for: Creating and validating cis-regulatory maps of tissue-specific gene expression regulation
Source: Nucleic Acids Res. 2014 Sep 8;42(17):11000–10. doi: 10.1093/nar/gku801 (PMC4176179; doi:10.1093/nar/gku801)
Supplement: SUPPLEMENTARY DATA [file supp_42_17_11000__index.html]

Creating and validating cis-regulatory maps of tissue-specific gene expression regulation — Creating and validating cis-regulatory maps of tissue-specific gene expression regulation — SUPPLEMENTARY DATA 

# Creating and validating cis-regulatory maps of tissue-specific gene expression regulation

## SUPPLEMENTARY DATA

**Files in this Data Supplement:**

- SUPPLEMENTARY DATA
