# Supplementary material for: Creating and validating cis-regulatory maps of tissue-specific gene expression regulation
Source: Nucleic Acids Res. 2014 Sep 8;42(17):11000–10. doi: 10.1093/nar/gku801 (PMC4176179; doi:10.1093/nar/gku801)
Supplement: SUPPLEMENTARY DATA [file supp_gku801_nar-01886-n-2014-File035.pdf]

Supplement to "Creating and validating cis-regulatory maps of tissue-specific gene expression regulation"

Timothy R. O'Connor and Timothy L. Bailey

August 20, 2014

1 Methods

1.1 Building correlation-based regulatory maps

To build a correlation-based regulatory map we require histone modification data (e.g., ChIP-seq data) and expression data (e.g., CAGE) for a number of tissues. Since the links in the map are based on statistically significant correlations between histone marks at a CRM and expression at a TSS, the power of the approach can be expected to increase with the addition of more (independent) tissues and more (independent) types of histone data. As noted above, our approach may require histone data or CAGE (at intergenic regions) for the test tissue for CRM prediction in order to build the map. Using gene expression data for the test tissue in the cross-tissue correlation is not required, but using it will improve the quality of the map if it is available.

To describe our map construction process we will use the following additional definitions. We denote a set of CRM genomic regions by $\mathbf{D}$. Let the annotated TSS set in the genome for which we have expression data be $\mathbf{T}$. Let $\mathbf{C}$ be the set of tissues for which we have histone and expression data, which may or may not include the test tissue, and let $\mathbf{H}$ be the set of histone types for which we have data. Let $X^c(t)$ be a measure of the expression at a TSS $t \in \mathbf{T}$ in tissue type $c \in \mathbf{C}$. Let $H^{c,h}(d)$ be a measure of the presence of histone type $h \in \mathbf{H}$ at CRM $d \in \mathbf{D}$ in tissue type $c \in \mathbf{C}$. (See below for further details of these measures of expression and histone state.) Let $\mathrm{S}(t, d, h)$ be the p-value of the Pearson correlation coefficient (PCC) of expression at TSS t and the presence of a histone mark h at CRM d, where the correlation is computed across all the tissue types $c \in \mathbf{C}$.

We can now define our correlation-based cis-regulatory map as the set of links between TSSs and CRMs such that the above correlation is statistically significant (at significance level $0 < \theta < 1$) for a given histone $h \in \mathbf{H}$:

$$\mathrm{CorrMap}_h(\theta) = \left\{ < t, d > \middle| \mathrm{S}(t, d, h) \leq \theta \right\}, \tag{1}$$

where $\mathrm{S}(t, d, h)$ is as defined in the previous paragraph. Note that we define the p-value of a CRM-TSS pair to be 1 if the PCC cannot be computed due to lack of data or zero variance in either the histone or expression data (See below for particulars of these constraints). Fig. 1 illustrates our map-building process.

To compute the p-value of the PCC used in Eqn. **1**, $\mathrm{S}(t, d, h)$, we first compute the PCC between the expression and histone measures $X^c(t)$ and $H^{c,h}(d)$, respectively. We then convert this to a Z-score using the Fisher transform (1), and compute its (unadjusted) p-value assuming a standard normal null distribution.

1.2 Linear regression model of expression

To fully describe our regression model we use the following additional definitions. Let $\mathbf{B}$ be the set of transcription factors for which we have binding information in the test tissue, $c \in \mathbf{C}$. Let $\mathbf{P}$ be the matrix of measures of TF binding near TSS locations, where rows correspond to to TSSs and columns to TFs. The entry $p_{t,b}$ in this matrix represents a log-transformed measure of the binding of TF $b \in \mathbf{B}$ near the TSS $t \in \mathbf{T}$ in tissue $c \in \mathbf{C}$, $p_{t,b} = \log(P^{c,b}(t) + \delta)$. (We describe the TF-promoter binding measure $P^{c,b}(t)$ and δ below.) Similarly, let $\mathbf{E}$ be the matrix of log-transformed

measures of TF binding within the CRMs associated with each TSS. The entry $e_{t,b}$ in this matrix represents a measure of the binding of TF $b \in \mathbf{B}$ within the *set* of CRMs linked with TSS $t \in \mathbf{T}$ in the map, $e_{t,b} = \log(E^{c,b}(t) + \delta)$. Likewise, let $\mathbf{N}$ be the matrix of log-transformed measures of TF binding within the CRMs associated with each TSS from a map with sampled CRM-TSS links (described later) for the same set of TSSs as above and each entry is calculated as in $e_{t,b}$. We describe TF-CRM-set binding measure $E^{c,b}(t)$ later. Let $\mathbf{Y}$ be a vector of log-transformed measures of expression of each of the TSSs, where entry y_t is represents the level of expression at TSS $t \in \mathbf{T}$ in tissue $c \in \mathbf{C}$, $y_t = X^c(t)$.

We can now define our (log-)linear model of expression for promoter, correlation-mapped CRMs, and sample-mapped CRMs as

$$\mathbf{Y} = \mathbf{P}\beta_1 + \beta_0, \mathbf{Y} = \mathbf{E}\beta_2 + \beta_0', \mathbf{Y} = \mathbf{N}\beta_3 + \beta_0'', \tag{2}$$

respectively, where β_1, β_2, and β_3 are vectors that contain the per-TF weights and β_0, β_0', and β_0'' are offsets to be fit by regression. These "CRM-TSS" models assume that the transcriptional expression at TSSs ($\mathbf{Y}$) can be estimated by a linear function of binding by TFs near the TSS ("promoter-binding", $\mathbf{P}$) and binding of the same TFs in the associated CRMs ("enhancer-binding", $\mathbf{E}$ and $\mathbf{N}$).

1.3 Creating sampled CRM-TSS maps

To demonstrate that the correlation-based mapping method identifies truly regulatory CRM-TSS links we create a set of maps as a control whereby the only substantive difference with the correlation-based maps are *which links* are in the map. In relation to the correlation-based map for which it forms the control, these sampled maps have the following properties:

- The same TSS set
- The same number of links
- The same CRM set to select from
- A similar distribution of links/TSS
- A similar distribution of CRM position relative to the TSS

To acheive these properties we implement the following proceedure.

For a correlation-based map built using a particular CRM, histone, and expression source and omitting a particular test tissue, we divide the ±1Mbp region up- and downstream of each TSS into 20 bins with equal occupancy of the number of linked CRMs in the correlation-based map. We then put all remaining unmapped CRMs into these bins to ensure that we sample from the same set of CRMs that were considered in the correlation-based mapping (but were excluded from the map at a given link stringency). We then sample a set of links to CRMs evenly from these bins to produce a map with the same number of links and similar positional distribution of CRMs. Finally, in order to produce a similar distribution of links/TSS we first sample one link for each TSS randomly from these bins to ensure each TSS has a minimum of one link as in the correlation-based map. We then sample links randomly without respect to which TSS is involved which produces a similar distribution of links/TSS. Note that it is possible to deplete bins of CRMs which may prevent creation of a sampled map. For each 5,000 failures of this type we reduce the number of bins by 2 until we can create a sampled map.

1.4 Measuring regression model accuracy

We evaluate the accuracy of our new map-based expression model using the R^2 measure of explained variance. This measures the ability of the model to predict expression from TF binding by showing the mean squared error (MSE) of the regression model's predictions compared to the variance in expression:

$$R^2 = 1 - \text{MSE}(\hat{\mathbf{Y}})/\text{var}(\mathbf{Y}) \tag{3}$$

where $\hat{\mathbf{Y}}$ is the predicted expresion from the regression model, and $\mathbf{Y}$ is the expression vector.

1.5 Measures of expression, histone modifications, TF binding

We use ENCODE data for all of our experiments except for the FANTOM5 CRM regions. For each experiment we use ENCODE RNA-seq or CAGE data for expression, histone ChIP-seq for histone modifications, and TF ChIP-seq for TF binding in a particular set of tissues (Table 1**A**; see Supp. Tab. 2- 1 for a full list of the source files)

- **C** is the set of tissues.
- **H** is the set of histone modification types.
- **B** is the set of transcription factors.
- $X^c(t)$ is a measure of the expression at TSS t in tissue $c \in \mathbf{C}$, and is the FPKM assigned to TSS t by ENCODE RNA-seq data set, or RPM assigned to TSS t by ENCODE CAGE data set. We average the FPKM or RPM values if more than one is reported for a given TSS.
- **T** is the subset of ENCODE-defined TSSs that 1) show substantial expression in at least one tissue and, 2) show sufficient variability in expression across the tissues. To be precise,

$$\mathbf{T} = \Bigg\{ t \Bigg| \, (\exists c \text{ s.t. } X^c(t) \geq 2) \;\; \text{AND} \\ \left(\frac{3}{2} \min_c(X^c(t)) \leq \bar{X}(t) \leq \frac{1}{3} \max_c(X^c(t)) \right) \Bigg\}, \tag{4}$$

  where $\bar{X}(t) = \sum_c X^c(t)/||\mathbf{C}||$ is the average expression of TSS t across all tissues $c \in \mathbf{C}$.

- $H^{c,h}(d)$ is a measure of the presence of histone modification $h \in \mathbf{H}$ in tissue $c \in \mathbf{C}$ at CRM d. Each such measure is based on a single ENCODE histone ChIP-seq experiment, and we define it to be the maximum height of any declared ChIP-seq peak that overlaps the CRM region by at least one base-pair.
- **D** is the subset of the combined, non-overlapping set of ENCODE(2)-defined tissue-specific CRMs, or FANTOM5 (3)-defined CRMs where at least one histone measure is non-zero in some other cell type than the omitted cell line. To be precise,

$$\mathbf{D} = \left\{ d \Big| \left(\exists c \in \mathbf{C}, \exists h \in \mathbf{H} \text{ s.t. } H^{c,h}(d) > 0 \right) \right\}. \tag{5}$$

  Note that we do not predict CRMs in this work.

- $P^{c,b}(t)$ is a measure of the binding of TF b in tissue c to TSS t. We use the sum of the TF ChIP-seq peaks within a window +/- 250bp from the TSS.

$$P^{c,b}(t) = \sum_k B(k), \tag{6}$$

  where k iterates over all peaks of TF b, and $B(k)$ is the height of peak k. In the regression parameter $p_{t,b} = \log(P^{c,b}(t) + \delta)$, $\delta = 1$.

- $E^{c,b}(t)$ is a measure of the aggregate binding of TF b in tissue c to all of the map-associated CRMs of TSS t. We define it as the sum of the heights of all ChIP-seq peaks that overlap any of TSS t's CRMs by at least one base-pair,

$$E^{c,b}(t) = \sum_d \sum_k B(k), \tag{7}$$

  where d ranges over the set of CRMs linked to TSS t in the map, $\{d | \exists < t, d > \in \text{Map}(\theta)\}$, and k ranges over the set of peaks that overlap CRM d by at least one base-pair. In the regression parameter $e_{t,b} = \log(E^{c,b}(t) + \delta)$, $\delta = 1$.

All map construction, data processing, score calculation, and experiments were implemented in the `C#` language. Regression was performed in `R` statistical software using the `cv.lars` function of the `lars` package (4).

1.6 Ensuring the validation process is unbiased

As noted above, none of the data (expression or histone modification) used to build a map is used in its validation. However, if the expression data in the tissues used to create the map is highly correlated with that of the map tissue, this could bias our map validation procedure. We therefore computed the average PCC of the expression at each TSS in a map between the reference (map) tissue and each of the comparative tissues used to build the map. At high link stringency the average PCC is quite low (Table 1**B**) for all RNA sources except short RNA-seq expression data. We also took machine learning measures within our regression model to ensure that the training and testing data were independent.

The high number of TF features relative to the number of TSSs on which the model regresses can lead to overfitting. To prevent overfitting of our regression models, we use LASSO regression (5) that includes a penalty term in the model in Eqn. 2 of the form

$$\mathbf{Y} = \mathbf{E}\beta_2 + \beta_0 - \lambda(\beta_0 + \sum_k \beta_{2,k}) \tag{8}$$

The λ in the penalty term can then be tuned to the value that minimizes overfitting by training models over all values of λ on a subset of training data and testing the model fit on a test set. We use the `cv.lars` method from the `R` statistical package `lars` (4) to use 10-fold cross-validation to tune the λ parameter. All regression results reported use the this model accuracy (see below) from this tuning process unless otherwise noted.

Since the test set we use for the LASSO training tunes a parameter, the model accuracy we report may have some bias as the test data are used to select the best λ value. As such, we also train LASSO models using a training set to learn the regression model for all values of λ, use a separate validation set for selecting the best λ value, and another separate test set to report the model error. In practice, this value was found to be approximately the same as what we reported using only training and testing datasets (Supp.Fig. 8).

1.7 Merging ENCODE CRM sources

The ENCODE CRM regions come from binding active regions (BARs) in five different tissues (with counts): GM12878 (213,542), H1-hESC (175,828), HeLa-S3 (174,856), Hep-G2 (155,250), and K562 (176,112). To predict regulatory targets of these CRMs and validate using LASSO regression we need a single CRM set. This single set of CRMs active in different tissues allows us to identify in a given tissue active TF binding corresponding to active expression at the predicted gene target and low or absent TF binding corresponding to low or inactive expression at the predicted gene target. While ENCODE provides an amalgamated CRM set combining CRMs from these five tissues, they merged clusters of overlapping CRMs into a single CRM, losing the tissue-specific CRM locations (2). In order to preserve these locations, we instead create a CRM set that removes some overlapping CRMs in order to retain a single, non-overlapping set that uses only tissue-specific CRM locations. We used bedtools (6) to implement the following proceedure to ensure a non-overlapping CRM set.

1. Identify all distinct CRMs from set of CRMs and set them aside in the final CRM set
2. Identify all clusters of overlapping CRMs that remain.
3. From each cluster, discard the CRM that overlaps with most other CRMs in that cluster.
4. Return to step 1 using the remaining CRMs in the overlapping clusters.

We run these steps iteratively, initially using the union of all ENCODE CRM regions from all tissues and setting the input of the next iteration to be the non-distinct, non-discarded regions that remain after step 3. After 11 iterations, this process converged (i.e., there were no more CRMs after step 1), reducing the initial union of all 895,588 BAR regions to a subset of 553,910 non-overlapping distinct BAR regions. We use this CRM set for all predictions of regulatory targets of ENCODE CRMs.

1.8 Restricting CRM-TSS link length

We constrain our mapping to a ±1Mbp region around each TSS because longer distances do not appear to capture more information than that contained the ±1Mbp region. Specifically, we see that the density of the location of CRMs linked to TSSs tends to decrease over the first 500-750Kbp, but remain constant at longer distances (Supp. Fig. 2). This region of higher linked CRM density around the TSS implies that this region is enriched in CRM-TSS links that represent true biological relationships and are not simply discovered by random chance. By comparison, we see that this region of high density is not present in maps that are built using a different histone mark whose regression model poorly fit tissue-specific TF binding to tissue-specific expression in a test tissue (Supp. Fig. 3**B**). We thus infer that an even distribution of density of linked CRMs implies that there is no enrichment of CRM-TSS links representing true biological relationships and that these links are more likely to be identified by random chance. We examined mapping over a larger range (±5Mbp) and found an even distribution of all linked CRMs located outside the ±1Mbp region even when using a histone mark we have shown to identify true CRM-TSS links (Supp. Fig. 4). Therefore, it does not appear that searching distances larger than ±1Mbp provides any benefit to the mapping method because the CRM-TSS links at those distances have no enrichment in true biological relationships.

1.9 Functional enrichment of genes in a cis-regulatory map

To analyze the function of the gene targets in maps, we selected the genes in the map for test tissue GM12878 built using CAGE and H3K27ac as a representative set of genes. We performed two tests. First, we examined functional enrichment of the set of genes in the map at the highest link stringency using the DAVID tool, which uses the EASE score (7), a variant of the Fisher exact test.

We report any terms in the biological process and cellular component gene ontology (GO) categories with a corrected p-value $<10^{-2}$. Second, we looked to see if any differences existed between genes targeted by a single CRM and those targeted by multiple CRMs. We again used the DAVID tool to identify enriched GO terms with the same criteria as above, but using the singly- or multiply-connected genes as a background for the complementary gene list rather than the human genome as a whole.

1.10 Availability

The source code for our map creation approach and maps for the five cell types and four RNA expression types are available at `http://research.imb.uq.edu.au/t.bailey/supplementarydata/OConnor2013`.

ENCODE CRM Data Sources	
Cell Line	File
GM12878	http://encodenets.gersteinlab.org/metatracks/BAR_Gm12878_merged.bed.gz
H1-hESC	http://encodenets.gersteinlab.org/metatracks/BAR_H1hesc_merged.bed.gz
HeLa-S3	http://encodenets.gersteinlab.org/metatracks/BAR_Helas3_merged.bed.gz
Hep-G2	http://encodenets.gersteinlab.org/metatracks/BAR_Hepg2_merged.bed.gz
K562	http://encodenets.gersteinlab.org/metatracks/BAR_K562_merged.bed.gz
**FANTOM5 CRM Data Sources**	
http://fantom.gsc.riken.jp/5/datafiles/latest/extra/Enhancers/hg19_enhancers.bed.gz	

Table 1: **CRM prediction source files from ENCODE (2) and FANTOM5 (3).** A single tar archive of all data is also available at: http://encodenets.gersteinlab.org/metatracks/BAR_All_merged.tar.gz.

Expression Data Sources	
Long PolyA+	
Directory Name:	wgEncodeCshlLongRnaSeq/
Filename format:	wgEncodeCshlLongRnaSeq[CellLine]PapTranscriptGencV7.gtf.gz
Cell Lines	A549, Ag04450, Bj, Gm12878, H1hesc, Helas3, Hepg2, Hmec, Hsmm, Huvec, K562, Mcf7, Nhek, Nhlf, Sknshra
Long PolyA-	
Directory Name:	wgEncodeCshlLongRnaSeq/
Filename format:	wgEncodeCshlLongRnaSeq[CellLine]PamTranscriptGencV7.gtf.gz
Cell Lines	A549, Ag04450, Bj, Gm12878, H1hesc, Helas3, Hepg2, Hmec, Hsmm, Huvec, K562, Nhek, Sknshra
CAGE	
Directory Name:	wgEncodeRikenCage/
Filename format:	wgEncodeRikenCage[CellLine]CellPapTssGencV7.gtf.gz
Cell Lines	A549, Ag04450, Bj, Gm12878, H1hesc, Helas3, Hepg2, Huvec, K562, Mcf7, Nhek, Sknshra

Table 2: **URL references for all expression data used in this work.** All data sources reside in http://hgdownload.cse.ucsc.edu/goldenPath/hg19/encodeDCC/[DirectoryName].

Histone ChIP-seq Data Sources	
Filename format: wgEncodeBroadHistone[CellLine][Modification]StdPk.broadPeak.gz	
Gm12878	H2az, H3k27ac, H3k27me3, H3k36me3, H3k4me1, H3k4me2, H3k4me3, H3k79me2, H3k9ac, H3k9me3, H4k20me1
H1hesc	H3k27ac, H3k27me3, H3k36me3, H3k4me1, H3k4me2, H3k4me3, H3k9ac, H4k20me1
Helas3	H3k04me1, H3k27ac, H3k27me3, H3k36me3, H3k4me2, H3k4me3, H3k79me2, H3k9ac, H4k20me1
Hepg2	H2az, H3k04me1, H3k27ac, H3k27me3, H3k36me3, H3k4me2, H3k4me3, H3k79me2, H3k9ac, H4k20me1
K562	H2az, H3k27ac, H3k27me3, H3k36me3, H3k4me1, H3k4me2, H3k4me3, H3k79me2, H3k9ac, H3k9me1, H3k9me3, H4k20me1

Table 3: **URL references for all histone ChIP-seq data used in this work.** All data sources reside in http://hgdownload.cse.ucsc.edu/goldenPath/hg19/encodeDCC/wgEncodeBroadHistone/.

A

θ	CAGE	Poly A+	Poly A-
10^{-10}	0.011 ± 0.029	0.076 ± 0.090	0.159 ± 0.163
10^{-15}	0.015 ± 0.059	0.011 ± 0.044	0.053 ± 0.160
10^{-20}	0.016 ± 0.078	0.005 ± 0.049	0.057 ± 0.164

B

θ	CAGE	Poly A+	Poly A-
10^{-10}	0.007 ± 0.029	0.046 ± 0.099	0.034 ± 0.170
10^{-15}	0.007 ± 0.038	0.020 ± 0.067	0.035 ± 0.175
10^{-20}	0.004 ± 0.037	0.016 ± 0.075	0.033 ± 0.173

Table 4: **Independence of expression data from multiple cell lines.** Each column shows the average (± standard deviation) Pearson correlation coefficient (PCC) of the given type of RNA expression data between pairs of the test tissues and all other cell lines used in map generation. For each type of RNA data, the PCC was computed over the TSSs included in the map at the given link stringency (θ). Panels **A** and **B** show data for TSSs in cis-regulatory maps built using ENCODE and FANTOM5 CRMs, respectively.

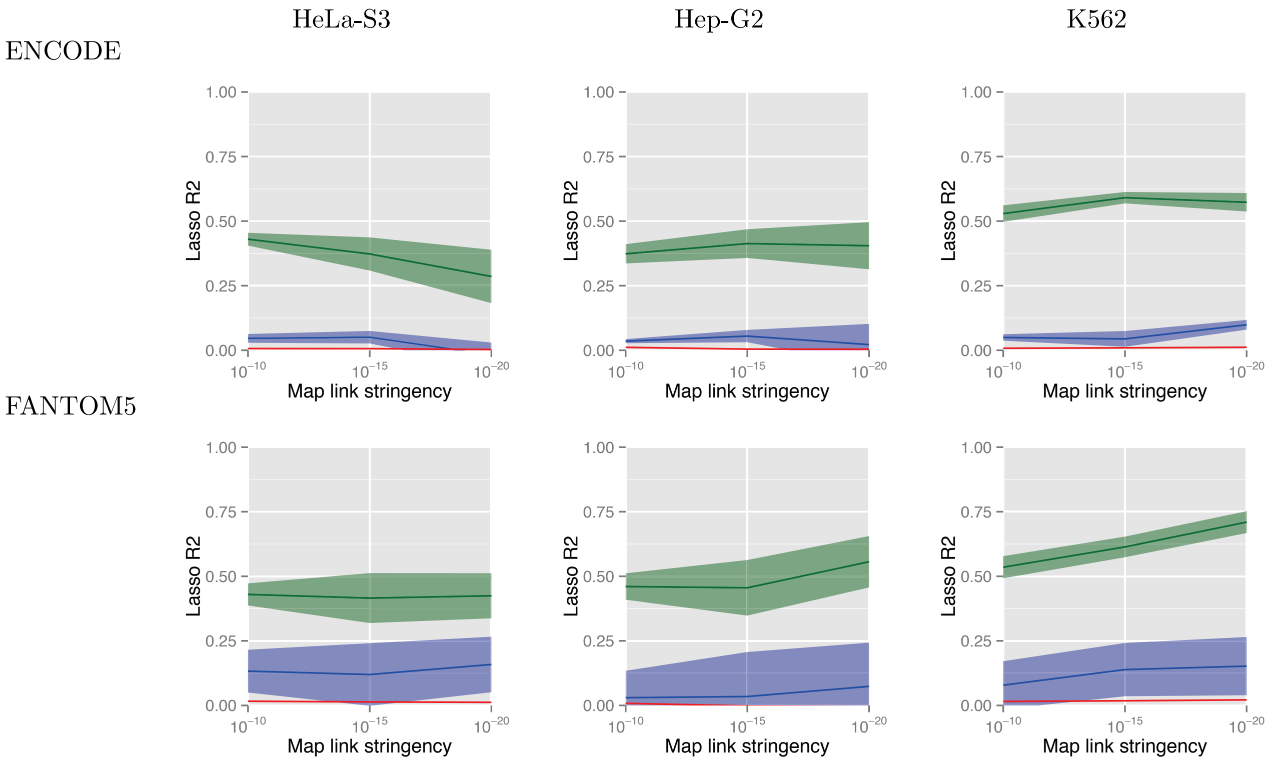

Figure 1: **Accuracy of H3K27ac-CAGE correlation cis-regulatory maps for three test tissues.** Each plot shows how the accuracy (explained variance, vertical axis) of models of gene expression based on cis-regulatory maps changes as a function of the link stringency (horizontal axis) of the expression-histone correlation of the CRM-TSS links in the map. The green and blue curves and areas show the mean and standard error of the LASSO R^2 for expression models using TF binding in either the promoters or CRMs of the map TSS set, respectively. The red curve shows the mean and standard error of the LASSO R^2 for 10 expression models based on 10 sampled control maps. The first column shows accuracy of models of CAGE expression in HeLa-S2 using ENCODE (top) and FANTOM5 (bottom) CRMs. The second column shows accuracy of models of CAGE expression in Hep-G2 using ENCODE (top) and FANTOM5 (bottom) CRMs. The third column shows accuracy of models of CAGE expression in K562 using ENCODE (top) and FANTOM5 (bottom) CRMs.

Transcription Factor ChIP-seq Data Sources	
Filename format: `wgEncodeSydhTfbs[CellLine][TFName]Pk.narrowPeak.gz`	
Cell Line	TF Names
Gm12878	Bhlhe40cIggmus, Brca1a300Iggmus, Cdpsc6327Iggmus, CfosStd, Chd1a301218aIggmus, Chd2ab68301Iggmus, Corestsc30189Iggmus, Ctcfsc15914c20Std, E2f4Iggmus, Ebf1sc137065Std, Elk112771Iggmus, ErraIggrab, Gcn5Std, Ikzf1iknuclaStd, Irf3Iggmus, JundIggrab, JundStd, MafkIggmus, MaxIggmus, MaxStd, Mazab85725Iggmus, Mxi1Iggmus, Nfe2sc22827Std, NfkbTnfaIggrab, NfyaIggmus, NfybIggmus, Nrf1Iggmus, P300Iggmus, P300bStd, P300sc584Iggmus, Pol2Iggmus, Pol2Std, Pol2s2Iggmus, Pol3Std, Rad21Iggrab, Rfx5200401194Iggmus, Sin3anb6001263Iggmus, Smc3ab9263Iggmus, Spt20Std, Srebp1Iggrab, Srebp2Iggrab, Stat1Std, Stat3Iggmus, Tblr1ab24550Iggmus, TbpIggmus, Tr4Std, Usf2Iggmus, WhipIggmus, Yy1Std, Znf143166181apStd, Znf274Std, Znf384hpa004051Iggmus, Zzz3Std
H1hesc	Bach1sc14700Iggrab, Brca1Iggrab, CebpbIggrab, Chd1a301218aIggrab, Chd2Iggrab, CjunIggrab, CmycIggrab, Ctbp2Ucd, Gtf2f1Iggrab, JundIggrab, MafkIggrab, MaxUcd, Mxi1Iggrab, Nrf1Iggrab, Rad21Iggrab, Rfx5200401194Iggrab, Sin3anb6001263Iggrab, Suz12Ucd, TbpIggrab, Usf2Iggrab, Znf143Iggrab, Znf274m01Ucd
Helas3	Ap2alphaStd, Ap2gammaStd, Baf155Iggmus, Baf170Iggmus, Bdp1Std, Brca1a300Iggrab, Brf1Std, Brf2Std, Brg1Iggmus, CebpbIggrab, CfosStd, Chd2Iggrab, CjunIggrab, CmycStd, Corestsc30189Iggrab, E2f1Std, E2f4Std, E2f6Std, Elk112771Iggrab, Elk4Ucd, Gcn5Std, Gtf2f1ab28179Iggrab, Hae2f1Std, Hcfc1nb10068209Iggrab, Ini1Iggmus, Irf3Iggrab, JundIggrab, MafkIggrab, MaxIggrab, MaxStd, Mazab85725Iggrab, Mxi1af4185Iggrab, NfyaIggrab, NfybIggrab, Nrf1Iggmus, P300sc584sc584Iggrab, Pol2Std, Pol2s2Iggrab, Prdm19115Iggrab, Rad21Iggrab, Rfx5200401194Iggrab, Rpc155Std, Smc3ab9263Iggrab, Spt20Std, Stat1Ifng30Std, Stat3Iggrab, TbpIggrab, Tcf7l2Ucd, Tcf7l2c9b92565Ucd, Tf3c110Std, Tr4Std, Usf2Iggmus, Zkscan1hpa006672Iggrab, Znf143Iggrab, Znf274Ucd, Zzz3Std
Hepg2	Arid3anb100279Iggrab, Bhlhe40cIggrab, Brca1a300Iggrab, CebpbForsklnStd, CebpbIggrab, CebpzIggrab, Chd2ab68301Iggrab, CjunIggrab, Corestsc30189Iggrab, ErraForsklnStd, Grp20ForsklnStd, Hnf4aForsklnStd, Hsf1ForsklnStd, Irf3Iggrab, JundIggrab, Maffm8194Iggrab, Mafkab50322Iggrab, Mafksc477Iggrab, MaxIggrab, Mazab85725Iggrab, Mxi1Std, Nrf1Iggrab, P300sc582Iggrab, Pgc1aForsklnStd, Pol2ForsklnStd, Pol2Iggrab, Pol2PravastStd, Pol2s2Iggrab, Rad21Iggrab, Rfx5200401194Iggrab, Smc3ab9263Iggrab, Srebp1InslnStd, Srebp1PravastStd, Srebp2PravastStd, TbpIggrab, Tcf7l2Ucd, Tr4Ucd, Usf2Iggrab, Znf274Ucd
K562	Arid3asc8821Iggrab, Atf106325Std, Atf3Std, Bach1sc14700Iggrab, Bdp1Std, Bhlhe40nb100Iggrab, Brf1Std, Brf2Std, Brg1Iggmus, Ccnt2Std, Cdpsc6327Iggrab, CebpbIggrab, CfosStd, Chd2ab68301Iggrab, CjunIggrab, CjunStd, CmycIggrab, CmycStd, Corestab24166Iggrab, Corestsc30189Iggrab, CtcfbIggrab, E2f4Ucd, E2f6Ucd, Elk112771Iggrab, Gata1bIggmus, Gata1Ucd, Gata2Ucd, Gtf2bStd, Gtf2f1ab28179Iggrab, Hcfc1nb10068209Iggrab, Hmgn3Std, Ini1Iggmus, JundIggrab, Kap1Ucd, MaffIggrab, Mafkab50322Iggrab, MaxIggrab, MaxStd, Mazab85725Iggrab, Mxi1af4185Iggrab, NelfeStd, Nfe2Std, NfyaStd, NfybStd, Nrf1Iggrab, P300Iggrab, P300sc584sc48343Iggrab, Pol2Iggmus, Pol2s2Iggrab, Pol2s2Std, Pol2Std, Pol3Std, Rad21Std, Rfx5Iggrab, Rpc155Std, Setdb1MnasedUcd, Setdb1Ucd, Sirt6Std, Smc3ab9263Iggrab, Tal1sc12984Iggmus, Tblr1ab24550Iggrab, Tblr1nb600270Iggrab, TbpIggmus, Tf3c110Std, Tr4Ucd, Ubfsc13125Iggmus, Ubtfsab1404509Iggmus, Usf2Iggrab, Xrcc4Std, Yy1Ucd, Zc3h11anb10074650Iggrab, Znf143Iggrab, Znf263Ucd, Znf274m01Ucd, Znf274Ucd, Znf384hpa004051Iggrab, Znfmizdcp1ab65767Iggrab

Table 5: **URL references for all TF ChIP-seq data used in this work.** All data sources reside in `http://hgdownload.cse.ucsc.edu/goldenPath/hg19/encodeDCC/wgEncodeSydhTfbs/`.

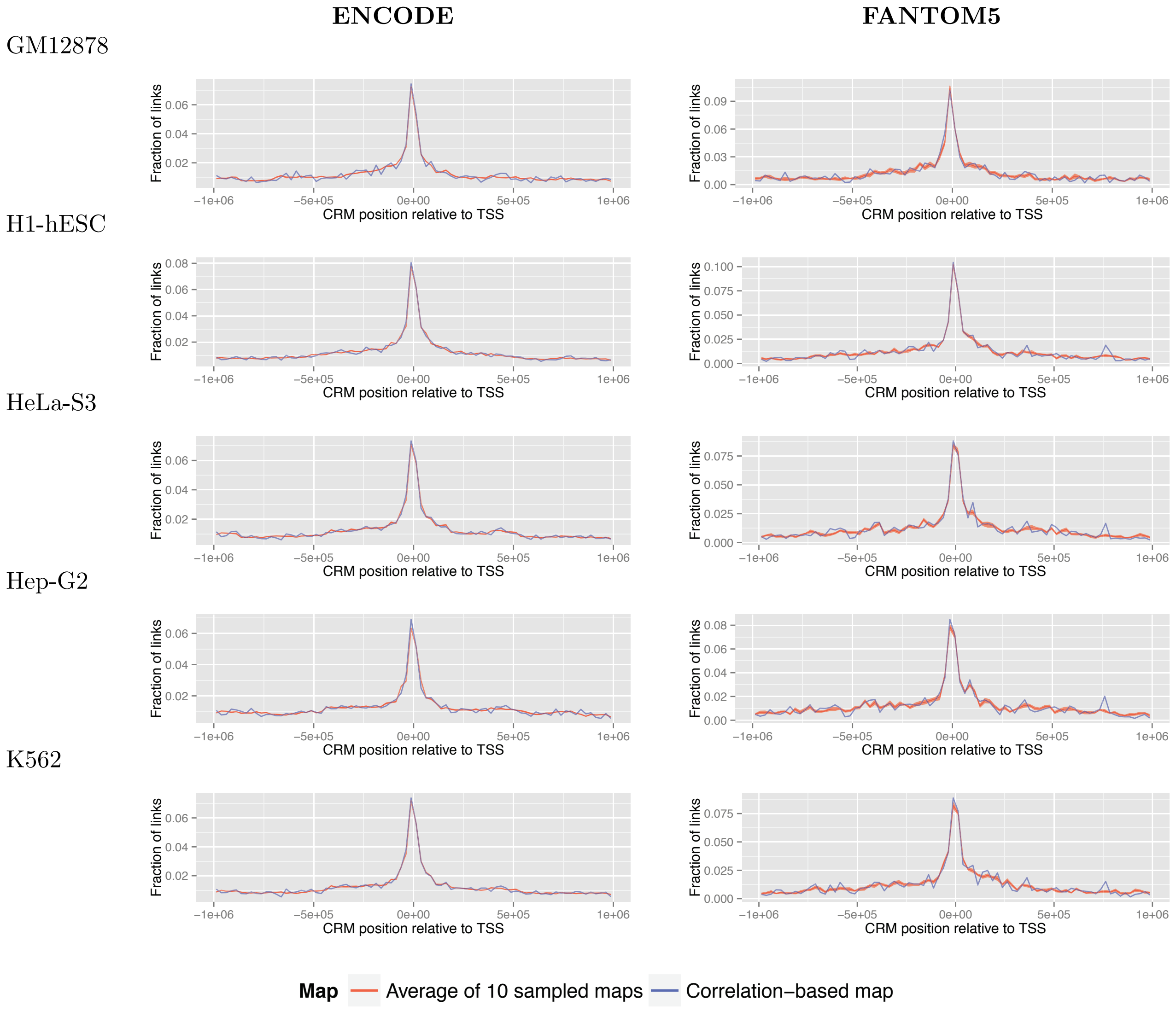

Figure 2: **Relative positions of CRMs linked to TSSs by H3K27ac-CAGE correlation for CRM source and omitted test tissue.** Each row shows histograms of the distribution of the position (bp) of linked CRMs in the sampled control and correlation-based cis-regulatory maps we derive using omitting the cell line named to the left of the histogram. The left column shows data using ENCODE as the CRM source and the right column shows data using FANTOM5 enhancers as the CRM source. The blue curve shows the fraction of linked CRMs at a given position relative to the TSS in the correlation map, in length bins of size 50Kbp. The red curve shows the mean and standard error of the fraction of linked CRMs (vertical axis) at a given position relative to the TSS (horizontal axis) in all 10 sampled control maps. For all maps, the link stringency is 10^{-20}.

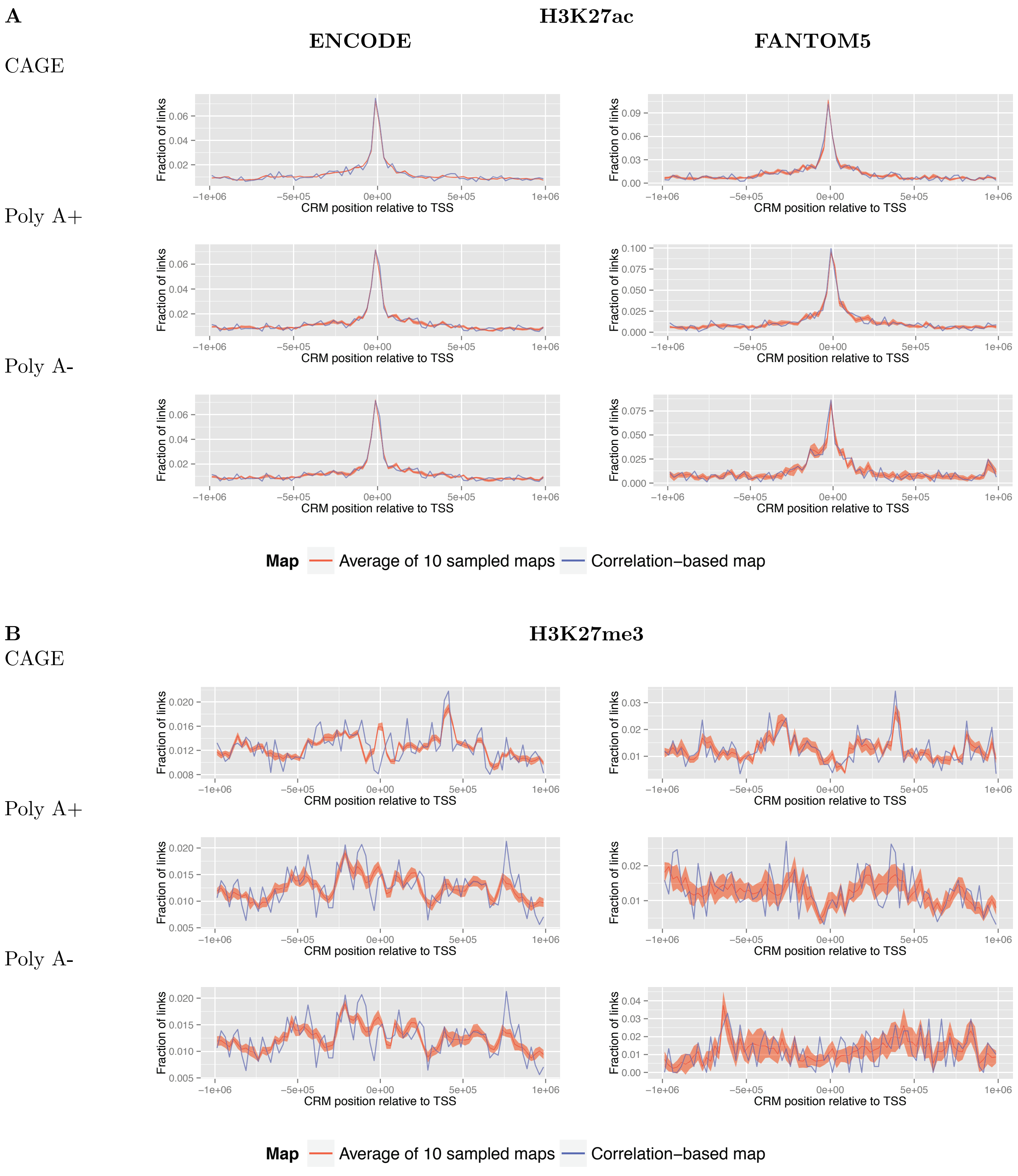

Figure 3: **Relative positions of CRMs to target TSSs in correlation-based and sampled control maps for different histone and expression sources.** Each row shows histograms of the distribution of the position (bp) of linked CRMs in the sampled control and correlation-based cis-regulatory maps omitting GM12878 that we derive using the RNA measure named to the left of the histogram. Tables **A** and **B** show data for maps using H3K27ac and H3K27me3 in the histone-expression correlation, respectively. The left column shows data using ENCODE as the CRM source and the right column shows data using FANTOM5 enhancers as the CRM source. The blue curve shows the fraction of linked CRMs at a given position relative to the TSS in the correlation map, in length bins of size 50Kbp. The red curve shows the mean and standard error of the fraction of linked CRMs at a given position relative to the TSS in all 10 sampled control maps. For all maps, the link stringency is 10^{-20}.

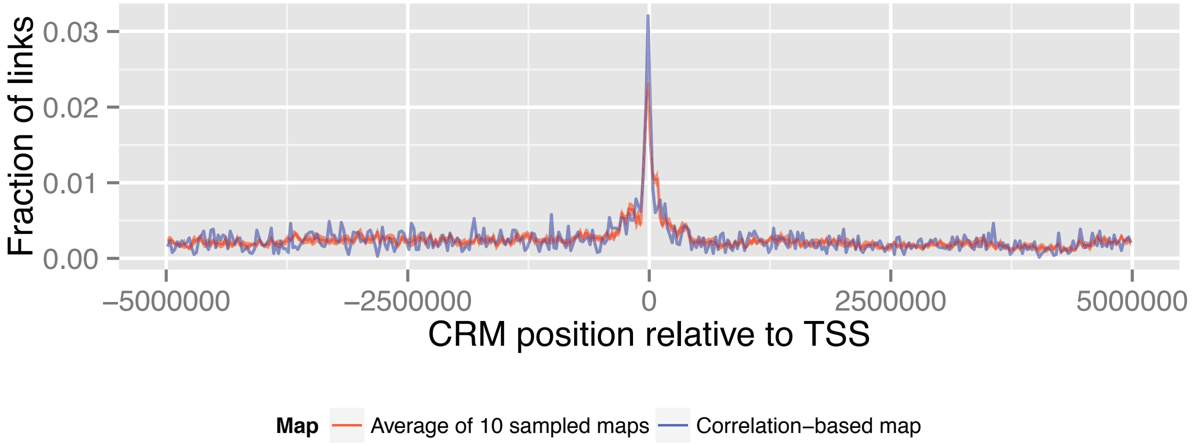

Figure 4: **Relative positions of CRMs to target TSSs mapped over a 5Mbp region.** The histogram shows the distribution of the position (bp) of linked FANTOM5 CRMs in the correlation-based cis-regulatory maps we derive using H3K27ac-CAGE correlation omitting data from GM12878. The blue curve shows the fraction of linked CRMs (vertical axis) at a given position relative to the TSS (horizontal axis) in the correlation map, in length bins of size 50Kbp. The red curve shows the mean and standard error of the fraction of linked CRMs at a given position relative to the TSS in all 10 sampled control maps. The link stringency is 10^{-20}.

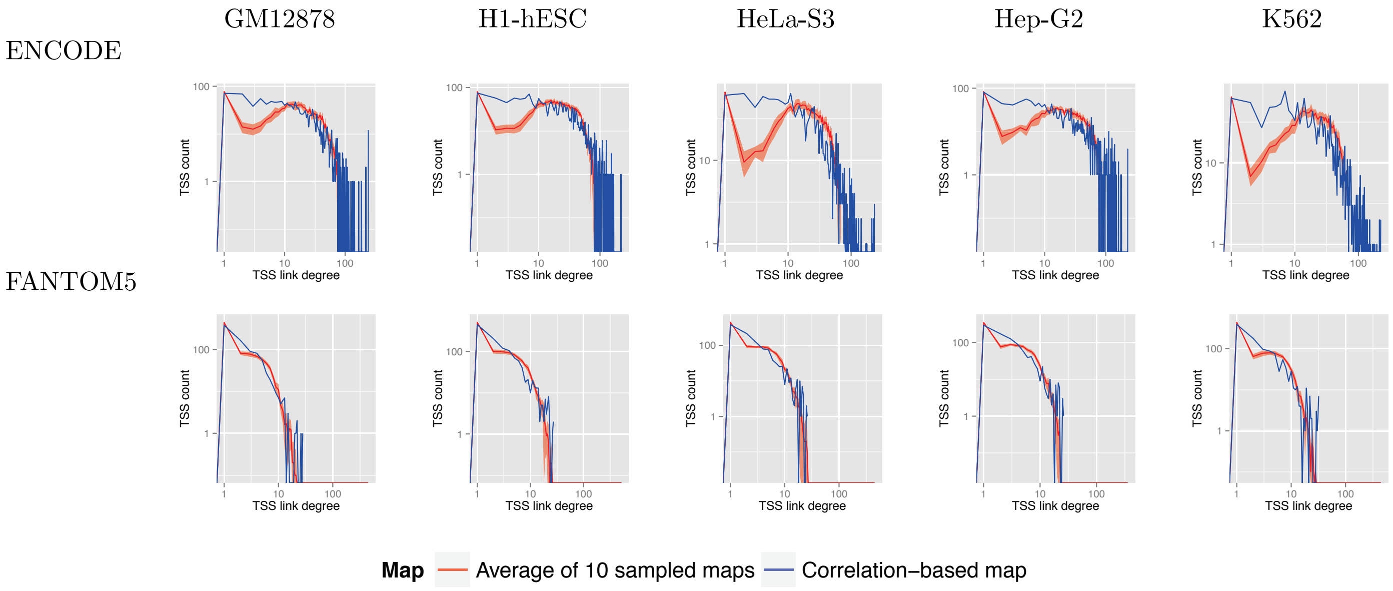

Figure 5: **TSS link count distribution for cell line and CRM source mapped using H3K27ac-CAGE correlation omitting GM12878.** Each row shows histograms of the distribution of TSS count (vertical axis) linked to a given number of CRMs (horizontal axis). Each column shows histograms for the cell line named above the histogram. Each row shows histograms for a given CRM source provided at the left. The blue curve shows the number of TSSs that are linked to a given number of CRMs for the correlation-based maps. The red curve shows the mean and standard error of the number of TSSs that are linked to a given number of CRMs across the 10 sampled maps. Map link stringency for all sources is 10^{-20}.

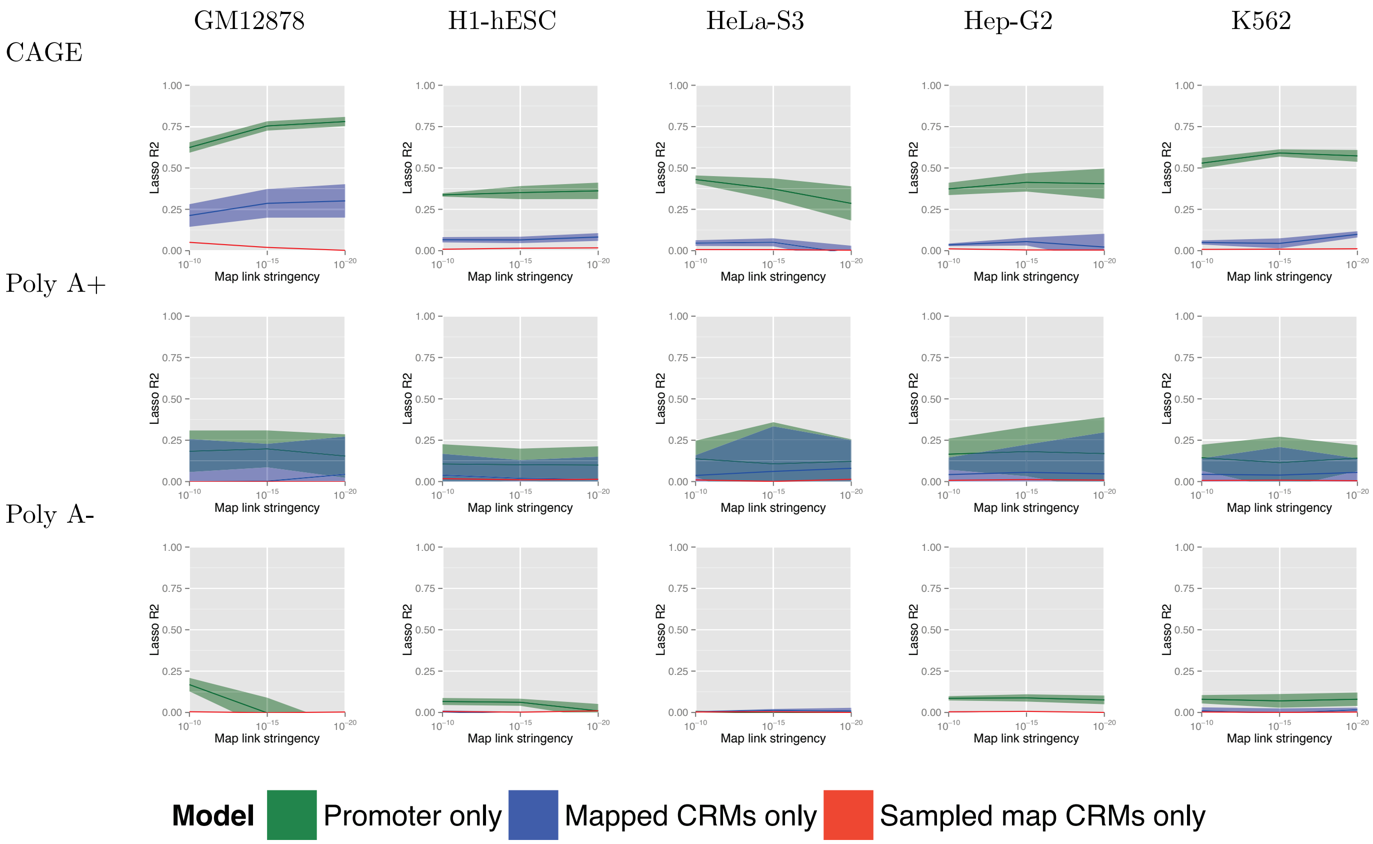

Figure 6: **LASSO regression for cis-regulatory maps correlating H3K27ac at ENCODE CRMs and different RNA expression sources for five different test tissues Each row shows plots of the of the LASSO fit of TF binding and expression (vertical axis) for cis-regulatory maps of increasing stringency (horizontal axis) for a given RNA expression source. Each column shows these plots for maps whose correlation omits the named test tissue. The blue curve shows the mean and standard error of the explained variance of expression at the TSS targets in a cis-regulatory map using TF binding in CRM regions that target that TSS. The red curve shows the mean and standard error of the explained variance of expression at the TSS targets in across 10 sampled maps using TF binding in CRM regions that target that TSS. The green curve shows the mean and standard error of the explained variance of expression at the TSS targets in a cis-regulatory map using TF binding in the promoter regions at the TSS.**

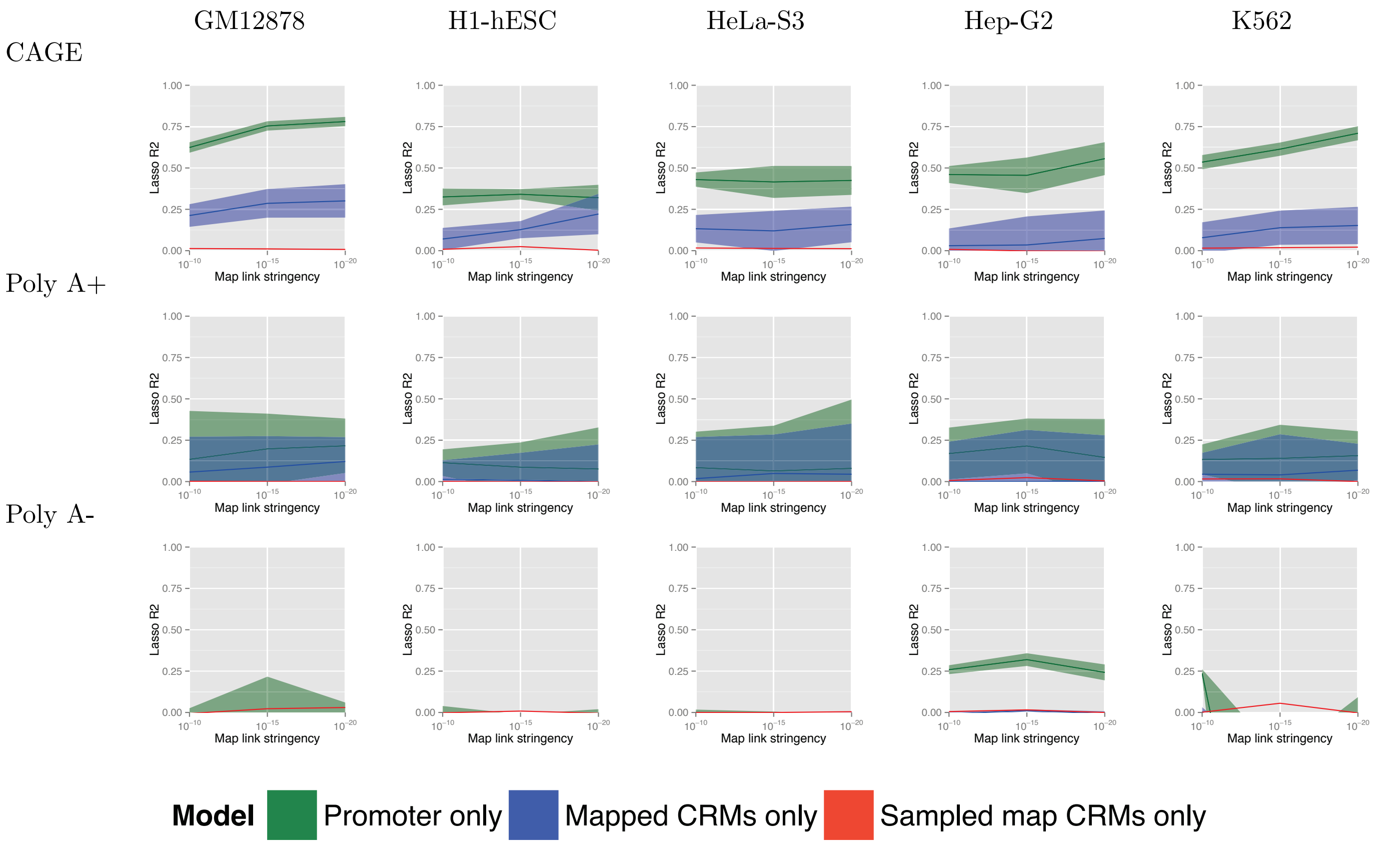

Figure 7: **LASSO regression for cis-regulatory maps correlating H3K27ac at FANTOM5 CRMs and different RNA expression sources using five different test tissues Each row shows plots of the of the LASSO fit of TF binding and expression (vertical axis) for cis-regulatory maps of increasing stringency (horizontal axis) for a given RNA expression source. Each column shows these plots for maps whose correlation omits the named test tissue. The blue curve shows the mean and standard error of the explained variance of expression at the TSS targets in a cis-regulatory map using TF binding in CRM regions that target that TSS. The red curve shows the mean and standard error of the explained variance of expression at the TSS targets in across 10 sampled maps using TF binding in CRM regions that target that TSS. The green curve shows the mean and standard error of the explained variance of expression at the TSS targets in a cis-regulatory map using TF binding in the promoter regions at the TSS.**

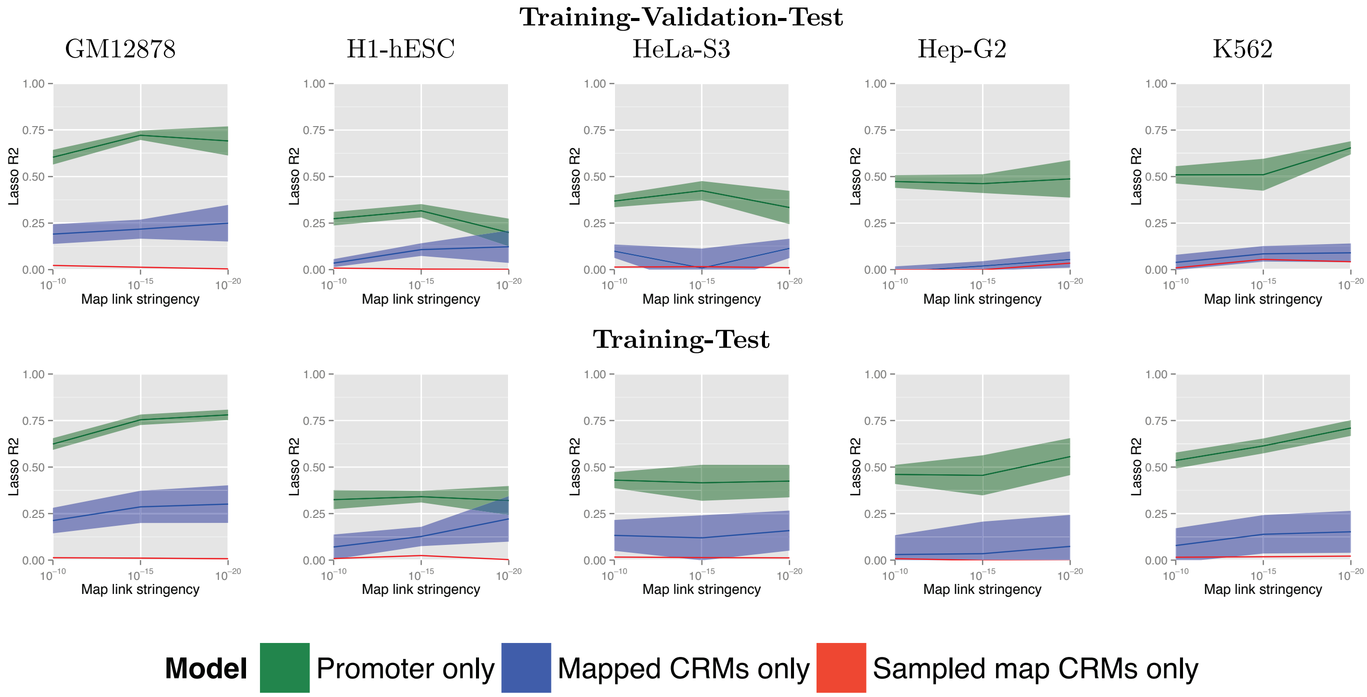

Figure 8: **LASSO regression for cis-regulatory maps using different testing paradigms.** Each row shows plots of the of the LASSO fit of TF binding and expression (vertical axis) for cis-regulatory maps of increasing stringency (horizontal axis) using the named testing paradigm. The "Training-Validation-Test" paradigm fits a LASSO model using the training set and tunes the λ parameter using the validation set and reports a cross-validated fit on the test set. The "Training-Test" paradigm fits a LASSO model using the training set, tunes the λ parameter using the test set, and reports the fit of the test set. Each column shows these plots for maps whose correlation omits the named test tissue. The blue curve shows the mean and standard error of the explained variance of expression at the TSS targets in a cis-regulatory map using TF binding in CRM regions that target that TSS. The red curve shows the mean and standard error of the explained variance of expression at the TSS targets in across 10 sampled maps using TF binding in CRM regions that target that TSS. The green curve shows the mean and standard error of the explained variance of expression at the TSS targets in a cis-regulatory map using TF binding in the promoter regions at the TSS.

A

Nearest neighbor count	TSSs with links	Links	CRMs	Links/TSS (median)	Median distance bp
	Overlap with correlation map (X)				
1	335	335	160	0.39 (0)	9,206
2	457	570	265	0.66 (1)	19,059
3	494	756	333	0.87 (1)	27,882
4	527	863	385	0.99 (1)	31,567
5	556	979	434	1.13 (1)	39,358
6	575	1115	477	1.28 (1)	47,821
7	611	1241	522	1.43 (1)	55,844
8	642	1341	566	1.54 (1)	63,149
9	651	1410	596	1.62 (1)	71,102
10	658	1473	617	1.70 (1)	78,623
X	869	2,530	1,075	2.91 (2)	207,970

B

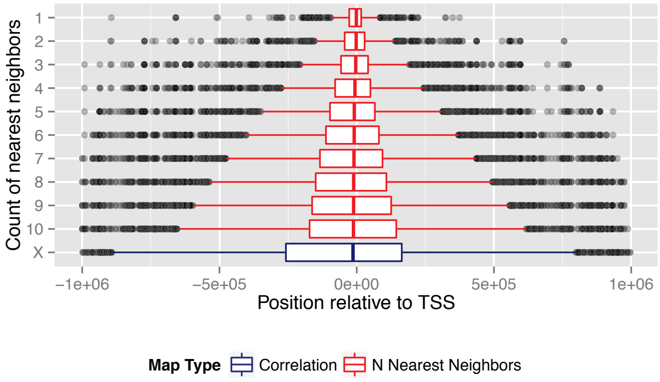

Figure 9: **Comparison of characteristics of cis-regulatory maps using H3K27ac-CAGE correlation with nearest neighbor (NN) maps.** The table in panel **A** shows the number of TSSs, links, and other statistics in the correlation-based map built using H3K27ac-CAGE correlation omitting test tissue GM12878 with a link stringency of 10^{-20} (NN count = X) and the overlap between that map and the nearest-neighbor map built with the given count (NN count $\in \{1, .., 10\}$). Additional statistics shown are the number of links present in both maps (Links) the median link length (median distance bp), the number of CRMs, and the average (and median) numbers of links per TSS (Links/TSS). Panel **B** shows box and whisker plots of the positions of CRMs relative to the TSS. The horizontal axis shows the distance upstream (-) or downstream (+) from the TSS, and the vertical axis shows data for each NN map (from 1 to 10 NN, red) and the correlation map (X, blue). The box shows the middle quartiles and the whiskers show the 95%ile with outliers shown in black.

A

Cell line	*TSS count in GRBs*	*CRM count in GRBs*	*Link count in GRBs*	*GRB count*	*GRB count with CRMs*	*GRB count with TSSs*	*GRB count with links*	*TSS count*	*CRM count*	*Link count*
**ENCODE**										
GM12878	26	418	390	240	14	49	13	1,527	18,004	39,807
H1-hESC	29	633	616	240	15	56	14	1,936	22,378	54,679
HeLa-S3	39	744	730	240	20	61	18	1,782	21,304	48,337
HepG2	39	666	709	240	22	55	22	1,470	19,473	44,266
K562	32	661	635	240	18	54	17	1,746	21,776	51,180
**FANTOM5**										
GM12878	12	40	57	240	8	15	5	868	1,075	2,529
H1-hESC	21	60	83	240	12	16	10	1,199	1,688	4,627
HeLa-S3	25	66	89	240	11	19	9	1,087	1,615	4,203
HepG2	23	62	88	240	13	18	11	944	1,530	3,854
K562	20	76	96	240	11	23	10	1,071	1,594	4,496

B

Cell line	*TSS count in SEs*	*CRM count in SEs*	*Link count in SEs*	*SE count*	*SE count with CRMs*	*SE count with TSSs*	*SE count with links*	*TSS count*	*CRM count*	*Link count*
**ENCODE**										
GM12878	26	166	118	257	5	41	5	1,527	18,004	39,807
H1-hESC	8	180	18	684	5	56	3	1,936	22,378	54,679
HeLa-S3	27	434	126	698	16	112	9	1,782	21,304	48,337
HepG2	7	59	35	497	3	28	1	1,470	19,473	44,266
K562	34	390	109	742	18	105	13	1,746	21,776	51,180
**FANTOM5**										
GM12878	16	37	30	257	4	18	4	868	1,075	2,529
H1-hESC	2	12	0	684	2	7	0	1,199	1,688	4,627
HeLa-S3	14	37	14	698	11	25	3	1,087	1,615	4,203
HepG2	6	5	5	497	2	5	1	944	1,530	3,854
K562	22	39	21	742	11	24	8	1,071	1,594	4,496

Table 6: **Overlap of genomic regulatory blocks (GRBs) or super enhancers (SEs) with cis-regulatory maps. Table A shows overlap between cis-regulatory maps and genomic regulatory blocks (GRBs) and table B shows the overlap between cis-regulatory maps and super enhancers (SEs). Each table shows the number of TSSs, CRMs, and links from a cis-regulatory map that are also located within a given set of genomic elements (GRBs, SEs). Each cis-regulatory map was built using H3K27ac-CAGE correlation using a link stringency of 10^{-20}. The first column names the cell line omitted from the correlation used to build the map (i.e., the test tissue). The next two columns (TSS/CRM count in GRBs/SEs) show the number of TSSs and CRMs, respectively, that are both in the cis-regulatory map and the given genomic elements. The column labeled "Link count in GRBs/SEs" shows the number of CRM-TSS links from the cis-regulatory map where both CRM and TSS are in a single region of the given type of genomic element. We also show the number of genomic regions examined (GRB/SE count) and indicate the number of these regions with a CRM, TSS, or CRM-TSS pair from the cis-regulatory map contained within them (GRB/SE count with CRMs/TSSs/links, respectively). Finally, we show the number of TSSs, CRMs, and links in the cis-regulatory map analyzed. Genomic regulatory blocks were taken from the UCNE database (8) (`http://ccg.vital-it.ch/UCNEbase/data/download/clusters/cluster_names.txt`). Super enhancers were taken from Hnisz *et al.* (9) using the corresponding super enhancer set for each of the given cell lines.**

ID	Term	Map genes with term	Map genes	Human genes with term	Human genes	Corrected *p*-value
**A**	**Cellular component**					
GO:0005576	extracellular region	89	272	2,010	12,782	8.33E-10
GO:0044421	extracellular region part	47	272	960	12,782	1.53E-5
GO:0005615	extracellular space	34	272	685	12,782	6.80E-4
GO:0031093	platelet alpha granule lumen	8	272	41	12,782	0.00128
GO:0031091	platelet alpha granule	9	272	56	12,782	0.00103
GO:0060205	cytoplasmic membrane-bounded vesicle lumen	8	272	44	12,782	0.00138
GO:0031983	vesicle lumen	8	272	46	12,782	0.00159
**B**	**Biological process**					
GO:0007398	ectoderm development	24	261	199	13,528	8.91E-9
GO:0008544	epidermis development	21	261	184	13,528	3.52E-7
GO:0009611	response to wounding	31	261	530	13,528	6.81E-5
GO:0007599	hemostasis	14	261	108	13,528	6.76E-5
GO:0042060	wound healing	18	261	191	13,528	5.95E-5
GO:0007596	blood coagulation	13	261	102	13,528	1.76E-4
GO:0050817	coagulation	13	261	102	13,528	1.76E-4
GO:0050878	regulation of body fluid levels	14	261	141	13,528	8.45E-4
GO:0030855	epithelial cell differentiation	13	261	137	13,528	0.00300
GO:0010817	regulation of hormone levels	13	261	151	13,528	0.00706
GO:0044058	regulation of digestive system process	5	261	11	13,528	0.00726
GO:0055088	lipid homeostasis	8	261	51	13,528	0.00837
GO:0002920	regulation of humoral immune response	5	261	12	13,528	0.00893

Table 7: **Cellular component and biological process gene ontology term enrichment of mapped genes. We report the gene ontology (GO) ID, term, and counts of genes with the given term in either a list of genes from a map or from the human genome as a whole. We report only those terms with a Benjamani-Hochberg-corrected *p*-value $<10^{-2}$. Genes analyzed come from the map built with H3K27ac-CAGE correlation omitting test tissue GM12878 with a link stringency of 10^{-20}. Table A shows the enriched cellular components and table B shows the enriched biological processes.**

A **LRRTM3**

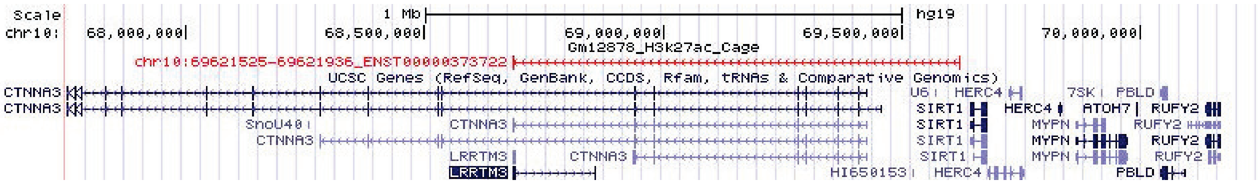

CRM location	PCC	p-value
chr10:69579652-69579872	-0.257	0.598
chr10:69582490-69582752	-0.167	0.737
chr10:69621525-69621936	1	0

B **MYCN**

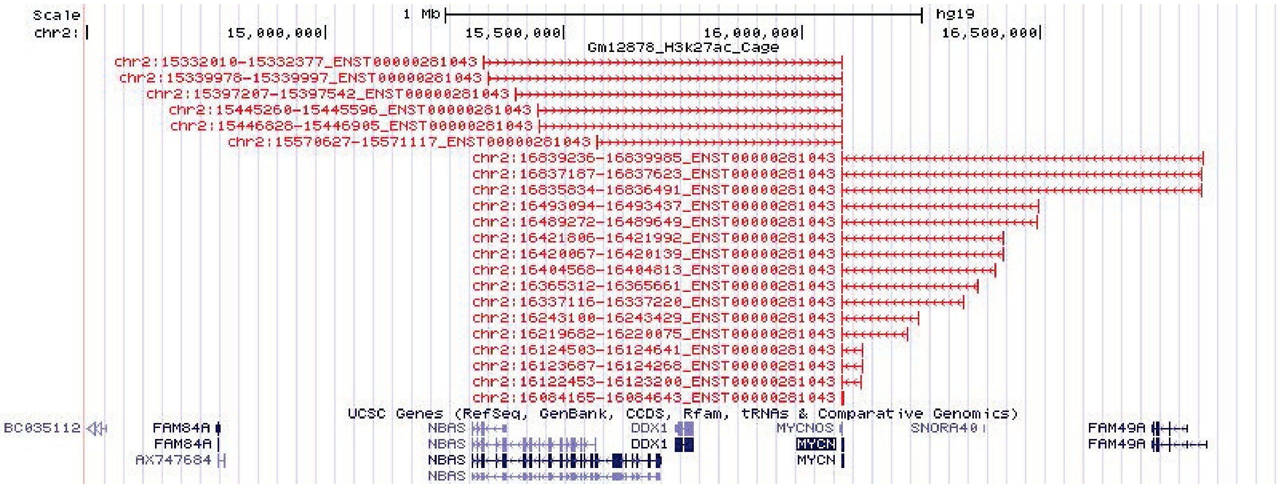

CRM location	PCC	p-value	CRM location	PCC	p-value
chr2:15309828-15310293	-0.202	0.681	chr2:15332010-15332377	0.99986	1.391E-21
chr2:15339978-15339997	0.99986	1.391E-21	chr2:15397207-15397542	0.99986	1.391E-21
chr2:15445260-15445596	0.99986	1.391E-21	chr2:15446828-15446905	0.99986	1.391E-21
chr2:15570627-15571117	0.99986	1.391E-21	chr2:15633080-15633249	-0.150	0.7625
chr2:15700375-15700622	-0.621	0.1461	chr2:15734291-15734400	-0.170	0.7312
chr2:15815563-15815811	-0.170	0.7312	chr2:16052794-16053077	-0.150	0.7635
chr2:16084165-16084643	0.99986	1.391E-21	chr2:16122453-16123200	0.99986	1.391E-21
chr2:16123687-16124268	0.99986	1.391E-21	chr2:16124503-16124641	0.99986	1.391E-21
chr2:16219682-16220075	0.99986	1.391E-21	chr2:16243100-16243429	0.99986	1.391E-21
chr2:16337116-16337220	0.99986	1.391E-21	chr2:16365312-16365661	0.99986	1.391E-21
chr2:16404568-16404813	0.99986	1.391E-21	chr2:16420067-16420139	0.99986	1.391E-21
chr2:16421806-16421992	0.99986	1.391E-21	chr2:16489272-16489649	0.99986	1.391E-21
chr2:16493094-16493437	0.99986	1.391E-21	chr2:16807698-16808010	0.132	0.7903
chr2:16827046-16827326	-0.170	0.7312	chr2:16831579-16831850	-0.0576	0.9092
chr2:16835049-16835704	0.229	0.6403	chr2:16835834-16836491	0.99986	1.391E-21
chr2:16837187-16837623	0.99986	1.391E-21	chr2:16839236-16839985	0.99986	1.391E-21
chr2:17042875-17043262	-0.170	0.7312			

Figure 10: **Example genes targeted by a single or multiple CRMs.** Each panel represents a region of human the genome around a specified gene as shown by the UCSC genome browser (10) and an accompanying table of all CRM-TSS pairs tested involving the specified gene. Panel **A** shows the three CRMs tested as putatively targeting the gene LRRTM3, one of which we predict to target that gene. Panel **B** shows the 33 CRMs tested as putatively targeting the gene MYCN, 22 of which we predict to target that gene. In each figure, the red track indicates predicted CRM-TSS links where each item shows an individual CRM-TSS link with the label indicating the genomic position of the CRM and the accession of the transcript at the TSS. The blue track shows the annotated genes in the given genomic region. The name of the specified gene is highlighted in blue. Links come from the map built with H3K27ac-CAGE correlation omitting test tissue GM12878 with a link stringency of 10^{-20}. In each table, each FANTOM5 CRM location within $\pm$1Mbp of the specified gene that meets our mapping criteria is given in the first column. The column "PCC" shows the Pearson Correlation Coefficient between H3K27ac at the CRM and the CAGE expression at the TSS of the specified gene across all tissues excluding GM12878. We also show the p-value associated with the PCC.

References

1. Fisher, R. A. May 1915 *Biometrika* **10(4)**, 507–521.

2. Yip, K. Y., Cheng, C., Bhardwaj, N., Brown, J. B., Leng, J., Kundaje, A., Rozowsky, J., Birney, E., Bickel, P., Snyder, M., and Gerstein, M. (2012) *Genome Biol* **13(9)**, R48.

3. Andersson, R., Gebhard, C., Miguel-Escalada, I., Hoof, I., Bornholdt, J., Boyd, M., Chen, Y., Zhao, X., Schmidl, C., Suzuki, T., Ntini, E., Arner, E., Valen, E., Li, K., Schwarzfischer, L., Glatz, D., Raithel, J., Lilje, B., Rapin, N., Bagger, F. O., Jrgensen, M., Andersen, P. R., Bertin, N., Rackham, O., Burroughs, A. M., Baillie, J. K., Ishizu, Y., Shimizu, Y., Furuhata, E., Maeda, S., Negishi, Y., Mungall, C. J., Meehan, T. F., Lassmann, T., Itoh, M., Kawaji, H., Kondo, N., Kawai, J., Lennartsson, A., Daub, C. O., Heutink, P., Hume, D. A., Jensen, T. H., Suzuki, H., Hayashizaki, Y., Mller, F., , F. A. N. T. O. M. C., Forrest, A. R. R., Carninci, P., Rehli, M., and Sandelin, A. Mar 2014 *Nature* **507(7493)**, 455–461.

4. Efron, B., Hastie, T., Johnstone, I., Tibshirani, R., et al. (2004) *The Annals of statistics* **32(2)**, 407–499.

5. Tibshirani, R. (1996) *Journal of the Royal Statistical Society (Series B)* **58**, 267–288.

6. Quinlan, A. and Hall, I. (2010) *Bioinformatics* **26**, 841–842.

7. Hosack, D. A., Dennis, G., Sherman, B. T., Lane, H. C., and Lempicki, R. A. (2003) *Genome Biol* **4(10)**, R70.

8. Dimitrieva, S. and Bucher, P. Jan 2013 *Nucleic Acids Res* **41(Database issue)**, D101–D109.

9. Hnisz, D., Abraham, B. J., Lee, T. I., Lau, A., Saint-Andr, V., Sigova, A. A., Hoke, H. A., and Young, R. A. Nov 2013 *Cell* **155(4)**, 934–947.

10. Kent, W. J., Sugnet, C. W., Furey, T. S., Roskin, K. M., Pringle, T. H., Zahler, A. M., and Haussler, D. Jun 2002 *Genome Res* **12(6)**, 996–1006.
